# Kaposi's Sarcoma-Associated Herpesvirus K-Rta Exhibits SUMO-Targeting Ubiquitin Ligase (STUbL) Like Activity and Is Essential for Viral Reactivation

**DOI:** 10.1371/journal.ppat.1003506

**Published:** 2013-08-22

**Authors:** Yoshihiro Izumiya, Keisuke Kobayashi, Kevin Y. Kim, Mamata Pochampalli, Chie Izumiya, Bogdan Shevchenko, Don-Hong Wang, Steve B. Huerta, Anthony Martinez, Mel Campbell, Hsing-Jien Kung

**Affiliations:** 1 Department of Dermatology, University of California Davis (UC Davis) School of Medicine, UC Davis Comprehensive Cancer Center, Sacramento, California, United States of America; 2 Department of Biological Chemistry and Molecular Medicine, UC Davis School of Medicine, UC Davis Comprehensive Cancer Center, Sacramento, California, United States of America; 3 Department of Basic Pathology, National Defense Medical College, Namiki, Tokorozawa, Saitama, Japan; 4 National Health Research Institutes, Taipei, Taiwan; University of Southern California Keck School of Medicine, United States of America

## Abstract

The small ubiquitin-like modifier (SUMO) is a protein that regulates a wide variety of cellular processes by covalent attachment of SUMO moieties to a diverse array of target proteins. Sumoylation also plays an important role in the replication of many viruses. Previously, we showed that Kaposi's sarcoma-associated herpesvirus (KSHV) encodes a SUMO-ligase, K-bZIP, which catalyzes sumoylation of host and viral proteins. We report here that this virus also encodes a gene that functions as a SUMO-targeting ubiquitin-ligase (STUbL) which preferentially targets sumoylated proteins for degradation. K-Rta, the major transcriptional factor which turns on the entire lytic cycle, was recently found to have ubiquitin ligase activity toward a selected set of substrates. We show in this study that K-Rta contains multiple SIMs (SUMO interacting motif) and binds SUMOs with higher affinity toward SUMO-multimers. Like RNF4, the prototypic cellular STUbL, K-Rta degrades SUMO-2/3 and SUMO-2/3 modified proteins, including promyelocytic leukemia (PML) and K-bZIP. PML-NBs (nuclear bodies) or ND-10 are storage warehouses for sumoylated proteins, which negatively regulate herpesvirus infection, as part of the intrinsic immune response. Herpesviruses have evolved different ways to degrade or disperse PML bodies, and KSHV utilizes K-Rta to inhibit PML-NBs formation. This process depends on K-Rta's ability to bind SUMO, as a K-Rta SIM mutant does not effectively degrade PML. Mutations in the K-Rta Ring finger-like domain or SIM significantly inhibited K-Rta transactivation activity in reporter assays and in the course of viral reactivation. Finally, KSHV with a mutation in the Ring finger-like domain or SIM of K-Rta replicates poorly in culture, indicating that reducing SUMO-conjugates in host cells is important for viral replication. To our knowledge, this is the first virus which encodes both a SUMO ligase and a SUMO-targeting ubiquitin ligase that together may generate unique gene regulatory programs.

## Author Summary

Protein modification by SUMO (small ubiquitin-like modifier), like phosphorylation, is now considered to be an important biochemical signal involved in nearly all cellular processes. Not surprisingly, it is also implicated in viral replication and host immune response. Timely turning on and off of SUMO signaling on viral and host proteins are important for virus to advance its replication. We previously described the identification of a viral SUMO E3 ligase encoded by Kaposi's sarcoma-associated herpesvirus (KSHV), which couples SUMO to recipient proteins. Here we report the discovery of a SUMO-targeting E3 ubiquitin ligase (STUbL) like function, also encoded by this virus. K-Rta preferentially degrades sumoylated proteins such as PML (promyelocytic leukemia) which negatively regulates viral replication. KSHV K-Rta is well recognized as a strong transcriptional factor and a trigger for viral reactivation. Recombinant KSHVs defective in reducing cellular SUMO conjugates are significantly compromised in their reactivation activity. Our finding not only uncovers a novel function of the transcriptional factor, K-Rta, but also points to the importance of dynamic regulation of the SUMO environment in herpesvirus replication.

## Introduction

Sumoylation (small ubiquitin-like modification) is now recognized as a universal signal transducer, rivaling phosphorylation. It affects nearly all cellular processes including transcription, RNA processing, DNA replication, DNA repair and chromosome segregation [1]–[7]. In a manner similar to the recognition of phosphorylated tyrosine by SH2 domain containing proteins, SUMO signals are engaged by proteins carrying a SIM (SUMO interactive motif) [8]–[11]. The SUMO/SIM interaction serves to propagate the cellular signals, which are initiated by SUMO conjugation to protein targets catalyzed by E3 SUMO ligases. There are only a handful of cellular SUMO ligases reported (e.g., PIAS family proteins, RanGAP, TRIM family proteins) [12]–[14]. The SUMO signal is terminated by SUMO deconjugation via SUMO specific proteases (i.e., SENP1, 2, 3, 5 and 6) [15]. Another way of SUMO signal attenuation whereby SUMOylated proteins are targeted for degradation has recently been described. RNF4, a SUMO-targeting ubiquitin ligase (STUbL), recognizes SUMO-2/3 (or poly-SUMO) conjugated proteins and targets them for ubiquitin-mediated degradation. A known RNF substrate is SUMO-PML (promyelocytic leukemia), a molecule involved in the formation of PML-nuclear bodies and implicated in the cellular antiviral response and tumor suppression [16]–[19].

Given the importance of sumoylation in cellular signaling, it is not surprising that viruses have evolved strategies to manipulate SUMO-signaling processes. Many viral proteins are themselves sumoylated [20]–[22]. There are also viral proteins which directly target the cellular SUMO machinery. A prime example is the avian adenovirus gene product Gam1, which interacts with and inhibits cellular SUMO E1 activating enzyme, resulting in the activation of many cellular genes [23]–[25]. We previously reported the identification of the first viral E3 SUMO ligase, K-bZIP, encoded by Kaposi's sarcoma-associated herpesvirus (KSHV) [14]. Here we report that KSHV also encodes an ubiquitin ligase, which preferentially targets SUMO-modified proteins.

Strong evidence suggests that KSHV is the causative agent for Kaposi's sarcoma, Castleman's disease and primary effusion lymphoma (PEL) [26]–[33]. Like other herpesviruses, the onset of viral lytic replication begins with the transactivation of immediate early and early genes, and the entry into latency involves the shut-down of the expression of the viral lytic genes. K-Rta and K-bZIP are two major viral transcriptional factors expressed very early after infection as a bi-cistronic transcript, and the gene products are involved in the modulation of viral transcription and replication. K-Rta is a strong transactivator which alone, is able to reactivate latent viral chromosomes and trigger the cascade of viral lytic replication [34], [35]. Our previous studies showed that K-Rta is able to potentially transactivate 35 KSHV promoters in transient reporter assays, and is recruited to promoters during lytic replication [36]. By contrast, K-bZIP is a SUMO-dependent transcriptional repressor, which associates with and attenuates K-Rta's transcriptional activity [20], [37]. In such a way, K-Rta activity is dynamically regulated during the viral life cycle, which may in part account for the temporal regulation of early and late viral genes.

Dynamic sumoylation is important during herpesvirus replication. Assembly of PML-NBs, a “mobile storage house” site for SUMO, depends on the sumoylation of PML and other components such as Daxx, SP100, and ATRX [38]–[41]. In addition, chromatin can be an integral part of PML-NBs. For example, a particular class of PML-NBs exists in tumor cells that maintain their telomeres without telomerase activity by a process referred to as alternative lengthening of telomeres (ALT). Recent studies identified that ALT is caused by the genomic mutations of Daxx or ATRX, both of which are localized in PML-NBs in a SUMO and SIM dependent manner [9], [42], [43]. Interestingly, an ATRX-Daxx complex was recently identified as a histone chaperone, which specifically deposits the histone H3 variant, H3.3 [44]–[46], raising the possibility that PML-NBs function as a center of epigenetic regulation.

Although the functions of PML-NBs in herpesvirus replication remain elusive, multiple herpesviruses have evolved ways to disassemble or disperse PML-NBs at the early phase of lytic infection. For example, HSV ICP0, a ubiquitin ligase [47] and CMV IE1 [48], [49] are able to degrade PML-NBs. On the other hand, EBV BZLF1 competes with PML for sumoylation and it disperses PML-NBs when EBV BZLF1 is overexpressed [22]. Taking into account that PML-NBs are IFN-inducible, PML-NB induction may be a part of host interferon response. Transcriptional factors residing in PML-NBs, such as Daxx and Sp100 functions have been shown to negatively regulate viral transcription [17], [50], [51], because these proteins recruit histone deacetylases as well as protein methylases [52]–[54]. In accord with this, Cuchet-Lourenco et al showed that PML-NBs were recruited to HSV-1 genomes after infection, and SUMO modification and SIMs of PML, Sp100, and hDaxx were found to be important in formation of a repressive complex on the genome. Importantly, the recruitment of PML-NBs to the genome was associated with viral gene repression, thus recruitment of PML-NBs represents one cellular defense mechanisms against invading pathogen DNA [55]. Thus, the inhibition of PML-NBs formation in early infection might be necessary to allow the viral replication/transcription to proceed efficiently *in vivo*. Since the formation of PML-NBs depends on sumoylation of PML, and its degradation depends largely on STUbL activity as exemplified by RNF4 [56], [57], modulation of sumoylation would help regulate the abundance of PML-NBs. Accordingly, HSV-1 encodes a STUbL-like protein, ICP0, which preferentially targets SUMO-modified proteins for degradation [58]. ICP0 protein degrades PML proteins by both SUMO dependent and independent mechanisms [58], [59]. HSV-1 recombinants with diminished STUbL-like function showed drastic reductions in viral reactivation [58]. A similar phenotype was also seen in Varicella-Zoster virus infection (VZV), in which the SUMO-binding function of ORF61 is important for PML-NBs disruption in both *in vitro* and in skin xenografts in a SCID mice model [60]. In this report, we show that KSHV K-Rta, an immediate-early protein, exhibits a STUbL-like function, which counteracts SUMO-chain formation.

## Materials and Methods

### Cell culture

Human embryonic kidney epithelial 293 cells and 293T cells were grown in monolayer culture in Dulbecco's modified Eagle medium supplemented with 10% fetal bovine serum (FBS) in the presence of 5% CO_2_. The K-Rta-inducible, TREx-K-Rta BCBL-1 cell line, generated by Nakamura et al. [34], was cultured in RPMI1640 supplemented with 15% FBS, 100 µg/ml of blasticidin (Invitrogen), and 100 µg/ml of hygromycin (Invitrogen). The Flag and HA-tagged SUMO-2 expression cassettes were introduced into TREx-K-Rta BCBL-1 and generated the stably expressing cells, TREx-K-Rta BCBL-1/FH-SUMO-2. Dual inducible TREx-Flag-Ubc9/Ubc9C93S-K-Rta cells that express both K-Rta and the SUMO conjugation enzyme Ubc9 or a catalytically inactive mutant, Ubc9C/S, were generated as described previously [61]. TREx-Flag-K-Rta Wt or mutant inducible 293 cells were generated similarly by co-transfecting with a Flpe recombinase expression plasmid and pcDNA5FRT/TO Flag-K-Rta plasmid. The TREx-293 cells were selected with 200 ug/mL of hygromycin for 2 to 3 weeks after co-transfection.

### Plasmids

Plasmids encoding the full-length K-Rta and K-Rta mutants were cloned into pcDNA3.1 (Invitrogen) as previously described [37]. K-Rta SIM-binding mutants were generated by a series of site-directed mutagenesis using the pcDNA3.1 K-Rta plasmid as a template. Full-length cDNAs of Sumo-1, Sumo-2, Sumo-3 and Ubc9 were amplified from BCBL-1 total RNA by RT-PCR and cloned into the *Cpo*I site of pcDNA-Flag or pGEX modified vector. Our SUMO protein sequences are identical with those previously reported (3) and these plasmids have been described previously [20]. Ubc9 C93S was generated by site-directed mutagenesis, and cloned into the pcDNA5FRT/TO vector upstream of the IRES-K-Rta fragment. A detailed map and the dual inducible transfer vector are available upon request. The PML and PMLΔSUMO expression vectors were generous gifts from Dr. Hsiu-Ming Shih (Academic Sinica, Institute of Biomedical Science, Taiwan).

### Immunoblot analysis

Immunoblotting was performed as described previously [37]. For detecting SUMO-modified proteins, the cells were washed twice with PBS and lysed with SUMO-lysis buffer (50 mM Tris-HCl [pH 6.8], 2% SDS, 10% glycerol, 20 µM N-ethylmaleimide [Sigma]) supplemented with a protease inhibitor cocktail (Roche). The lysates were immediately boiled for 10 min and centrifuged, and the supernatant was used for immunoblot analysis.

### Antibodies

Anti-K-Rta antibody was previously described [62]. Anti-HA antibody (Covance), anti-GAPDH (Upstate), anti-promyelocytic leukemia protein (PML) mouse monoclonal antibody (PG-M3; Santa Cruz), anti-β-tubulin (Sigma), anti-Flag M2 antibody (Sigma), anti-actin (Sigma), anti-PCNA (ab18197; Abcam), anti-MDC1 (ab11169; Abcam), and anti-53BP1 (ab36823; Abcam) were commercially obtained. Immunoblotting of PML was performed by using anti-PML antibody (ab72137; Abcam).

### Recombinant protein purification

Recombinant proteins were expressed and purified from either bacteria or insect cells as described previously [20]. The purity and concentration of protein was estimated by SDS-PAGE with BSA as a standard. Artificial SUMO-2 chains, which consist of four tandem SUMO peptides were generated by ligating PCR products encoding ΔN11-SUMO2 as described elsewhere [57]. One difference is that the most N-terminal SUMO-2 peptide was engineered to encode the residues of full length of SUMO-2 (residue 2-92).

### Immunofluorescence assays (IFA)

IFA were performed as described previously. Briefly, cells were fixed with 3.7% formaldehyde and permeabilized with 1% SDS and 1% Triton-X100 treatment in PBS for 5 min each at room temperature. Primary antibody was incubated overnight in 2% BSA-PBS. Secondary antibody (Alexa Fluor 488 or Alexa Fluor 555 conjugated antibodies; Invitrogen) was incubated for 1 hour at room temperature after washing with PBS four times. Slides were mounted with Anti-fade gold (Invitrogen).

### *In vitro* SUMO conjugation

*In vitro* SUMO conjugation reactions were done as described previously [20]. The E1 (SAE1/2), E2 (Ubc9), His-SUMO-1, 2, and 3 were commercially obtained (Boston Biochem). SUMO reactions were performed according to manufacturer's protocol. SUMO-chains were generated using GST-SUMO-2 or -3 bound to glutathione beads as substrates. The SUMO conjugation reactions were performed for 6 hours at 37°C. The products of the SUMO reactions were subjected to SDS-PAGE after washing with high a salt buffer, and chain formation was confirmed with Coomassie staining. In order to eliminate SUMO enzymes that were non-covalently bound to SUMO-chain beads, SUMO-chain beads were washed with the high salt (0.5 M NaCl) buffer containing 10% glycerol. The SUMO-chain beads were used for pull-down analyses as well as ubiquitin conjugation reactions. Effects of K-Rta in SUMO conjugation was examined by adding purified K-Rta proteins (100 nM) in the SUMO conjugation reaction. The reaction was performed for 3 hours at 37°C. One microgram of purified PML was also used as a substrate and incubated with SUMO conjugation reaction components in the presence or absence of K-Rta protein (100 nM). For PML SUMO conjugation, SUMO reactions were performed for 1 hour.

### *In vitro* ubiquitin conjugation

Ubiquitin E1 (UBE1), E2 (Ubc5a), and HA-ubiquitin were obtained from a commercial source (Boston Biochem). Poly-SUMO-2 chain (Boston Biochem) or GST-SUMO-2 was used as a substrate. Ubiquitin conjugation reactions were performed using final enzyme concentrations of E1 (25 nM), E2 (50 nM), E3 [K-Rta or K-Rta mutant (30 nM or 120 nM) [as indicated in figure legends] in a buffer (50 mM Tris-HCl [pH 7.5], 1 mM ATP, 1 mM MgCl_2_) for 30 min to 3 hour at 37°C.

### *In vitro* protein interaction analysis

Purified protein was incubated with GST-proteins bound on beads. The SUMO-2 chain was purchased from commercial source (Boston Biochem). The GST-pull down assays were performed as described previously [37].

### Northern blotting

Total RNA was prepared with TRIzol (Invitrogen) as recommended by the manufacturer. Total RNA (10 µg/lane) was separated on 1.2% agarose-formaldehyde gels and transferred to nylon membranes. The RNA was immobilized on the membrane by drying at RT for 1 h and UV cross-linking. DNA probes were prepared from total genomic DNA of BCBL-1 cells by PCR. DNA fragments purified from agarose gels were radiolabeled with [α^32^-P]dATP using a Strip-EZ DNA kit (Ambion) as recommended by the supplier. Hybridization was performed with ULTRAhyb buffer (Ambion) as recommended by the supplier.

### Reporter assays

A dual luciferase reporter system (Promega) was used to quantify promoter activity. 293T cells were transfected in 12-well plates. Three hundred nanograms of reporter were cotransfected with 150 nanograms of expression plasmid in each well. Two days after transfection, the cells were washed with PBS, and incubated with 250 µl of passive lysis buffer provided by the manufacturer. Reporter constructs were previously described [36]. A *Renilla* TK expression vector (10 ng/well) served as an internal control. Ten microliters of cell lysate per well were assayed using a Glo-Max instrument (Promega) according to the manufacturer's recommendations. At least three independent assays were carried out for each experiment.

### Preparation of recombinant KSHV

Mutagenesis of a bacterial artificial chromosome containing the entire KSHV genome (BAC-16) was performed by using a recombineering system (http://recombineering.ncifcrf.gov). The bacterial strains and vectors used in the recombineering procedure were obtained from the NCI. The host strain for lambda-mediated recombination was a BAC-16 SW105 transformant, and KSHV BAC16 was generous gift from Dr. Jung (University of Southern California). A K-Rta targeting vector was constructed as presented in Figure S1. The targeting vector contains a *Hind*III/*Bgl*II fragment which covers the K-Rta coding region as well as the partial K8 coding region. The kanamycin cassette which is flanked by site-specific recognition (FRT) sequences for the FLPe recombinase [63] was inserted into *Sal*I sites located between the K-Rta and K-bZIP coding regions of the targeting vector. The linearized targeting cassette contains homology arms of 2.1 kb and 0.1 kb on the 5′ and 3′ side of the kanamycin resistance marker. The targeting cassette was released from the vector sequences by digestion with *Hind*III and *Bgl*II (Accession number NC_009333 nt.72848-75029). The fragment was gel purified and electroporated into induced (recombination +) SW105/BAC-16 cells. Kanamycin-resistant transformants were selected and analyzed by PCR for insertion of the targeting cassette. Two or three positive clones of each intermediate were then grown at 32°C with removal of the targeting cassette accomplished by arabinose induction of the FLPe recombinase. Kanamycin-sensitive clones were screened by PCR for removal of the targeting cassette. Clones positive by PCR for removal of the cassette were then further verified by sequencing of the targeted region of K-Rta. A similar approach was used for the K-Rta SIM mutants that were cloned into the targeting vector. The introduced Ring or SIM mutations were confirmed by direct sequencing of PCR-amplified targeted site and the integrity of each recombinant BAC was examined by restriction enzyme digestions and Southern blotting (Figure S1). Southern blotting was performed as described previously with K-Rta coding sequence as a probe [37].

### K-Rta wild-type revertant construct

K-Rta Ring mutant alleles in BAC-16 were reverted to wild-type using the recombineering protocols described above. For these constructions, the mutant K-Rta allele in the KSHV bacmid was replaced with wild-type K-Rta using the wt-K-Rta targeting cassette. Reversion of the K-Rta Ring-mutant allele to wild-type was confirmed by sequencing. 293T BAC stable cell lines containing revertant bacmids were then created as described below.

### Construction of BAC stable cell cultures

293T cells were transfected with 5 µg of recombinant BAC DNA using HEKfectin (Bio-Rad). After two days of culture, the cells were expanded to a single T175 flask and hygromycin (200 µg/ml) selection was started. After approximately two weeks, the hygromycin-resistant colonies that emerged from a single flask were trypsinized and pooled to establish each culture. Early passage pools were used throughout the experiments described herein.

## Results

### KSHV K-Rta affects general sumoylation of cellular proteins

**Figure 1:**
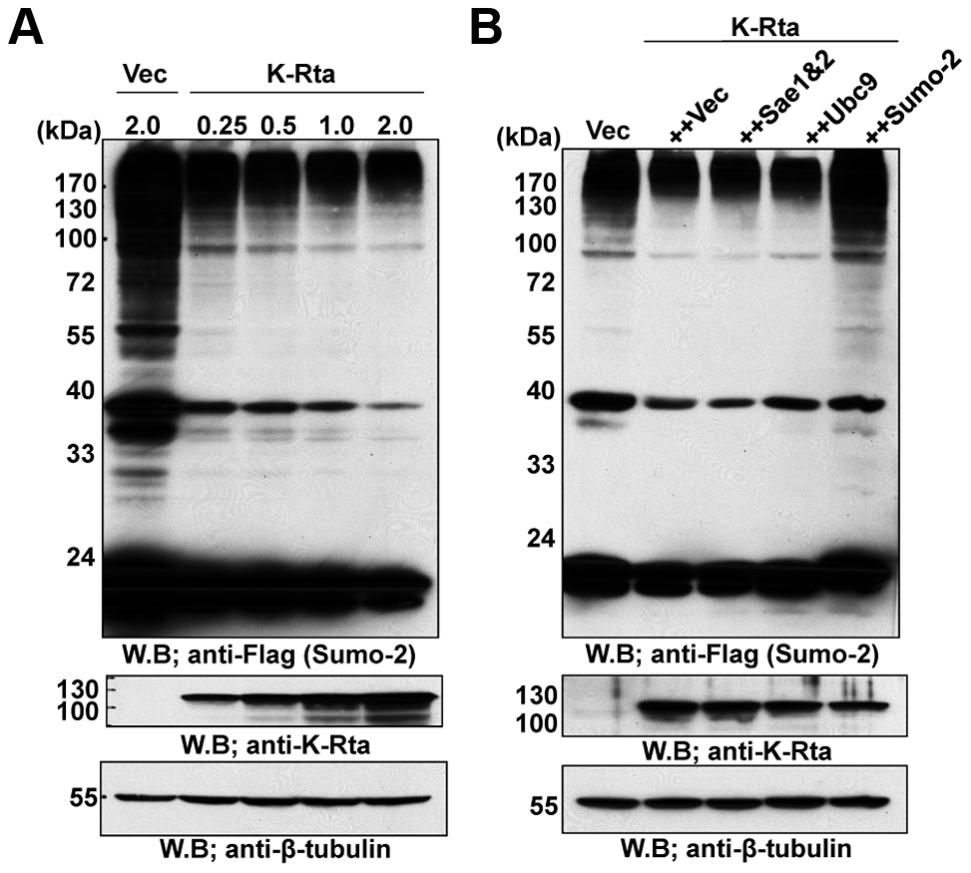
Degradation of SUMO by K-Rta. Plasmids expressing E1 (Uba2/Aos), E2 (Ubc9), SUMO-2, K-Rta or empty vector (Vec) were transfected into 293T cells. SUMO, K-Rta or tubulin was probed with respective antibody. **(A)** K-Rta reduced total SUMO-modified protein levels in a dose-dependent manner. **(B)** Overexpression of SUMO but not other SUMO enzymes recover the cellular SUMO-modified proteins.

KSHV encodes an immediate-early gene product, K-Rta, a potent transcriptional factor whose expression is essential for the initiation of viral replication and emergence from latency. Our previous work [36] and others' [64], [65] showed that K-Rta expression effectively switched KSHV episomes from a heterochromatin to an euchromatin state with concomitant loss of KAP-1, a process linked to de-sumoylation [61], [66], [67]. This activity of K-Rta, together with its ability to disrupt PML-NBs (see below), prompted us to examine the regulation of the SUMO pathway by K-Rta. First, a SUMO-2 expression vector was co-transfected with different amounts of K-Rta, and SUMO-modification of cellular proteins was examined by probing western blots with an antibody against SUMO. The SUMO-modified proteins are seen as a smeared ladder, due to the presence of multiple sumoylated proteins and the multimeric SUMO chains attached to these proteins (Figure 1A, Vec). The expression of K-Rta significantly reduced the sumoylation of cellular proteins in a dose-dependent manner (Figure 1A), suggesting that K-Rta may interact or interfere with the basic cellular SUMO-machinery. This reduction can be seen by transfection with as little as 250 nanograms of the K-Rta expression vector, which is one-eighth the quantity of transfected SUMO, suggesting a catalytic role of K-Rta in the removal of SUMO or sumoylated host proteins. In addition, the depletion of SUMO or SUMOylated proteins by K-Rta can be seen to associate with all three SUMO paralogues, SUMO-1, 2 and 3 (Figure S2).

Four possible scenarios can be envisioned to account for K-Rta's effects on cellular sumoylation; (i) interference with SUMO conjugation by inhibiting cellular SUMO E1, E2 or E3, as was the case of the adenoviral protein Gam 1, (ii) enhancement of SUMO removal by up-regulation of desumoylases such as SENPs, (iii) facilitation of the degradation of SUMO and SUMOylated proteins by SUMO-targeting ubiquitin ligase or STUbL induced by K-Rta, and finally, (iv) K-Rta has STUbL function. Our microarray data (not shown) with K-Rta inducible 293 cells did not reveal differences in expression levels of SENPs, which does not support scenario (ii). We next asked whether overexpression of E1, E2 or SUMO can overcome the K-Rta effects, resulting in the recovery of cellular SUMO-profile. As shown in Figure 1B, only over-expression of SUMO can rescue the SUMO-depletion phenotype induced by K-Rta.

### K-Rta targets SUMO for degradation

Based on the above results, we hypothesized that K-Rta targets SUMO for degradation either directly by serving as an ubiquitin ligase or by activating a cellular ubiquitin ligase which targets SUMO moieties. The cellular protein RNF4 and its family members were recently discovered as STUbLs which specifically target SUMO moieties and sumoylated proteins for degradation [3], [56], [57], [68]. Quantitative RT-PCR analysis did not reveal alteration of RNF4 expression in K-Rta expressing cells (data not shown), suggesting that the degradation activity may be a direct effect of K-Rta. Accordingly, we investigated whether K-Rta itself has a STUbL like function. To be classified as a STUbL, K-Rta should fulfill the following criteria: (i) possess ubiquitin (Ub) ligase activity (ii) contain a SIM domain(s) which recognizes a SUMO-moiety, and (iii) the Ub ligase activity preferentially targets SUMO and SUMOylated proteins for degradation. Criterion (i) has already been met by the studies of Yang et al. [69] and Yu et al. [70]. Here we address the other two measures.

### K-Rta contains SIMs and directly binds SUMO moieties

**Figure 2:**
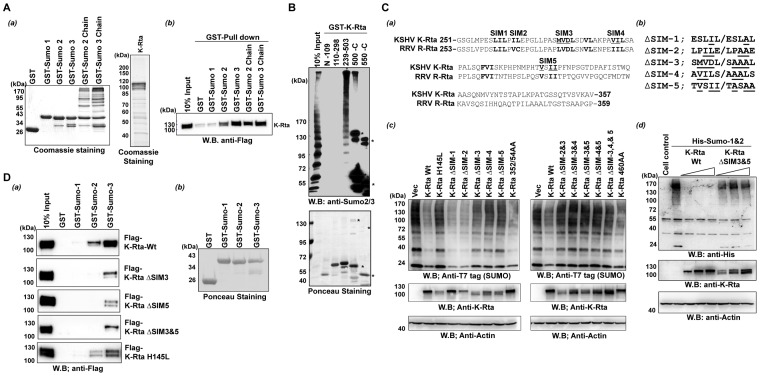
K-Rta physically associates with SUMO. (**A**) (a) Recombinant GST and GST-SUMO were purified from an *E. coli* expression system, and GST-SUMO chains were generated *in vitro*. Flag-K-Rta protein was purified from recombinant baculovirus infected Sf9 cells (right panel). (b)The GST-Pull down assay was performed by incubating GST, GST-SUMO-1, -SUMO-2, -SUMO-3, -SUMO-2 chain or –SUMO-3 chain with purified Flag-K-Rta. Association was detected by immunoblotting with anti-Flag antibody. (**B**) Mapping of SUMO-binding domain. The indicated K-Rta deletion protein was incubated with SUMO-2 chains, and the interaction was probed with an anti-SUMO monoclonal antibody (upper panel). The membrane was also stained with Ponceau to show amount of GST-K-Rta deletion protein on the membrane (bottom panel). Asterisks showed non-specific interaction between C-terminal K-Rta and IgG. (**C**) Generation of K-RtaΔSIM. (a) Multiple alignments between KSHV Rta and RRV Rta. Conserved hydrophobic clusters (putative SIM) are marked in bold letters. Mutations that showed decreased SUMO degradation are underlined. (b) Mutations introduced in this study are shown. (c) SUMO degradation by K-Rta mutants. Immunoblotting was performed after the transfection of indicated K-Rta mutant plasmids along with SUMO expression vector in 293T cells. Mutations at SIM-3, -4, or -5 impaired K-Rta SUMO degradation function. (d) K-Rta Wt but not K-RtaΔSIM degrades SUMO modified proteins. Increasing amounts of K-Rta Wt or K-RtaΔSIM3&5 (0.25, 0.5, or 1.0 ug) were cotransfected with SUMO. The SIM mutation completely abolished the SUMO degradation function of K-Rta. (**D**) (a) GST pull-down analyses with purified K-Rta Wt or K-Rta ΔSIM mutants are shown. The interaction was probed with anti-Flag antibody. (b) Ponceau staining shows the amount and purity of GST- or GST-SUMO proteins used in the assay.

To demonstrate direct binding, purified K-Rta and SUMO were prepared using baculovirus and *E. coli* expression systems respectively, and incubated together. A SUMO-chain was generated by performing *in vitro* SUMO-conjugation reactions, and GST, GST-SUMO, or GST-SUMO-chain was incubated with purified K-Rta (Figure 2A(a)). As shown in Figure 2A(b), K-Rta binds to GST-SUMO proteins, both monomeric and multimeric forms, but not GST alone, indicating that K-Rta is a SUMO-binding protein. Similar to KSHV K-bZIP [14], K-Rta bound SUMO-2 and -3 but not SUMO-1 in the binding buffer containing 250 mM NaCl. The reciprocal experiment, which involved the pull down of purified SUMO multimers by various deletion mutants of GST-K-Rta also confirmed the interaction between K-Rta and SUMO (Figure 2B). This experiment helped delineate the SUMO-binding region(s) of K-Rta to be localized between amino acids 239 to 503. Furthermore, K-Rta appears to prefer SUMO-multimers over monomers as revealed by the higher intensity of SUMO in the high- molecular- weight region pulled down by GST-K-Rta (as compared to the input lane) (Figure 2 A, B), which is similar to what was seen for RNF4 [57]. This suggests that heavily sumoylated proteins, likely SUMO2/3 conjugated, are preferentially bound by K-Rta for subsequent degradation. During course of experiments, we noticed that C-terminal portion of K-Rta strongly interacts with immunoglobulin, which generated background (shown as asterisks in Figure 2B).

### K-Rta contains SIMs

Inspection of amino acid sequence of the K-Rta SUMO binding domain showed several putative SIMs that each consist of a short hydrophobic core, often flanked by acidic residues. Alignments between RRV Rta and KSHV K-Rta showed conservation of several hydrophobic clusters (Figure 2C(a)). Accordingly, those hydrophobic residues were individually mutated to alanine (Figure 2C(b)), and K-Rta SIM mutants were screened by cotransfection with SUMO to examine effects on SUMO degradation (Figure 2C(c)). Although mutation of SIM1 or SIM2 did not affect the SUMO degradation function of K-Rta, mutations of SIM3, 4, or 5 significantly impaired SUMO degradation function. Similarly, mutations of hydrophobic residues outside of SIM clusters such as 352/54AA or 460AA did not alter SUMO and SUMO-mediated protein degradation function of K-Rta (Figure 2C and Figure S3A), which is consistent with the fact that the amino acid sequence outside of these SIM clusters was much less conserved between RRV and KSHV. These results were further confirmed by an independent experiment, in which we used different doses of K-Rta and the SIM 3&5 double mutant to assess the SUMO degradation function of K-Rta. The results showed that mutations of both SIM3 and SIM5 significantly impaired K-Rta SUMO degradation function (Figure 2C(d)). Finally, the SUMO-binding capability of several SIM mutants was examined by GST-pull down assays. In addition, the relative contributions of SIMs in SUMO binding were also examined after purifying K-Rta mutant proteins. The purified K-Rta proteins were incubated with purified GST-SUMOs. The interaction between K-Rta and SUMO was visualized by immunoblotting with Flag antibody. As shown in Figure 2D and Figure S3B, the single mutation present in either SIM3 or 5 was sufficient to decrease SUMO binding, and the combination of these mutations had only a marginal additional defect in SUMO binding relative to each individual mutant. The K-Rta Ring-mutant (K-Rta H145L), which still possessed SIM domains, bound SUMOs, indicating a specific interaction between K-Rta SIM domain and SUMOs. The fact that the SIM mutants still weakly interacts with SUMO-3 in our binding condition (250 mM NaCl), suggests that there might be other SUMO binding domain(s), which was missed in our experiments.

### The Ring-like domain of K-Rta mediates the depletion of sumoylated proteins

**Figure 3:**
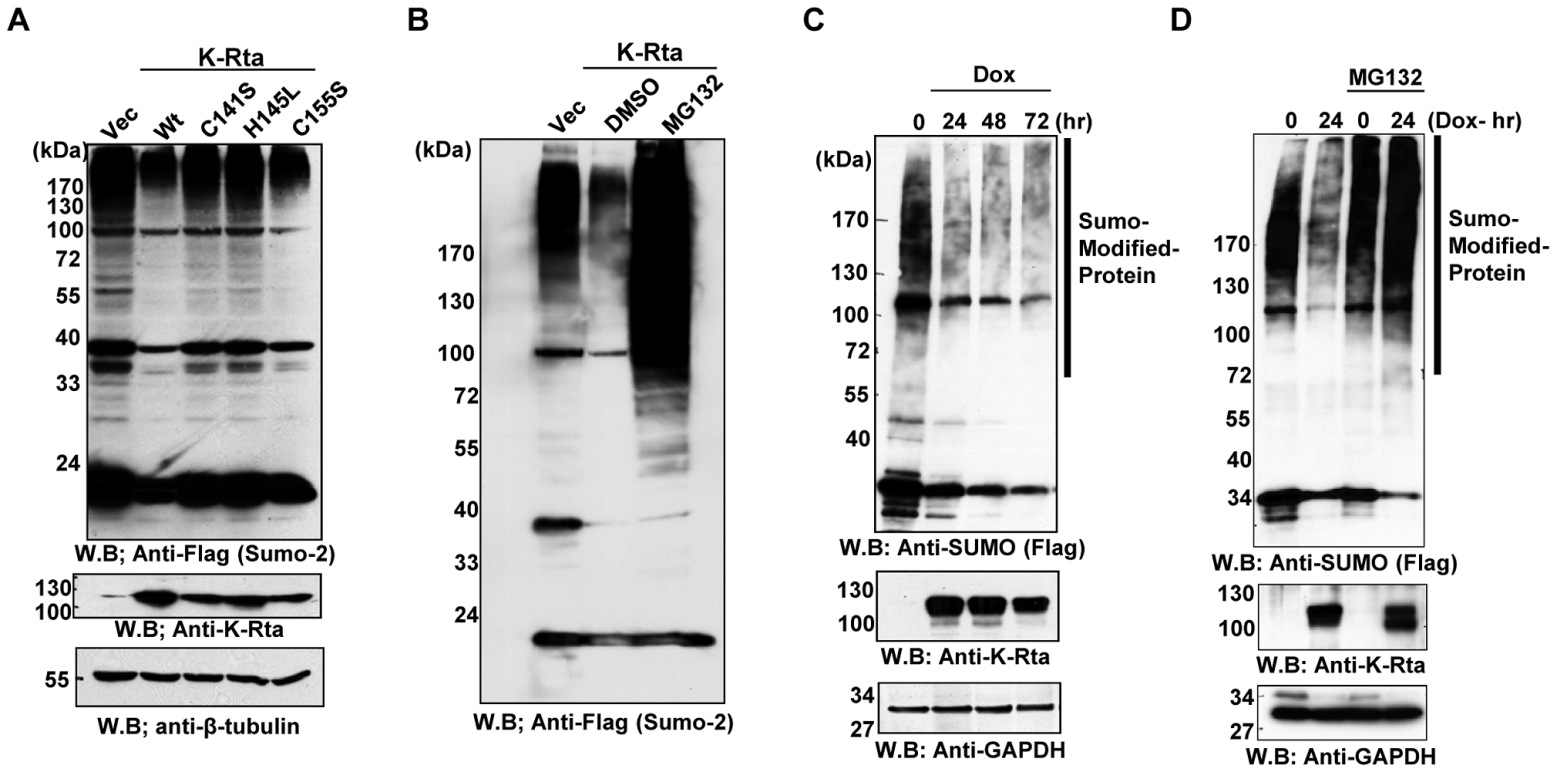
K-Rta Wt but not Ring-finger domain mutant can degrade SUMO-modified proteins. (**A**) Flag-SUMO and K-Rta wild type or mutant were cotransfected into 293T cells, and probed with indicated antibodies. The K-Rta Ring-like domain (C141, H145) is important for SUMO degradation. (**B**) MG132 recovered SUMO proteins from degradation. 24 hours after the K-Rta transfection, MG132 or DMSO (vehicle) was added into the culture media and cells were harvested after 12 hours of treatment. SUMO-modified proteins were probed with anti-Flag antibody. (**C**) SUMO degradation during KSHV reactivation in BCBL-1. After induction of K-Rta expression in TREx-K-Rta-BCBL-1 with doxycycline (Dox), SUMO-modified proteins were probed with anti-Flag antibody. K-Rta induction was confirmed with an anti-K-Rta antibody and GAPDH was served as the loading control. (**D**) Recovery of SUMO-modified proteins with MG132 in BCBL-1. KSHV reactivation was triggered by induction of K-Rta in BCBL-1 cells in either the presence or absence of MG132. The amount of SUMO-modified proteins was examined by immunoblotting with an anti-Flag antibody. The accumulation of SUMO-modified conjugates was evident by increments of higher molecular weight entities.

Previous work [69], [70] showed that K-Rta possesses ubiquitin ligase activity and that a Ring-like domain in K-Rta with conserved cysteines and histidines is critical for this activity [70]. To examine whether ubiquitin E3 ligase function is involved in SUMO depletion, K-Rta ubiquitin ligase mutants as well as a control mutant were prepared. Wild type and mutant K-Rta were transfected into 293T cells and the resulting cellular SUMO modification patterns were compared. Mutation of cysteine 141 or histidine 145 within the Ring domain significantly diminishes K-Rta's ability to deplete SUMO, whereas mutation of cysteine 155 outside the Ring-like domain had no effect (Figure 3A). This result suggests that the ubiquitin ligase activity of K-Rta is important for the depletion of SUMO. In agreement with this notion, a proteasome inhibitor, MG132, added 24 hours after K-Rta transfection, significantly restored the level of SUMO-2 modified proteins, suggesting proteasome dependence of K-Rta-mediated protein degradation (Figure 3B).

As a next step, we asked whether this phenomenon is also observed in naturally infected cells during reactivation. To increase the sensitivity of this assay, we have developed a BCBL-1 cell line with tagged-SUMO-2 stably transfected, and which expresses K-Rta in an inducible manner. As shown in Figure 3C, once K-Rta is induced in BCBL-1 cells, SUMO-2 is rapidly depleted, which is accompanied by viral reactivation. Depletion of SUMO-2 was again recovered in the presence of MG132 (Figure 3D) or epoxomicin (not shown). Taken together, these data demonstrated that K-Rta depleted SUMO in both an ubiquitin ligase and proteasome-dependent manner *in vivo*.

### K-Rta induces ubiquitylation of SUMO *in vitro*

**Figure 4:**
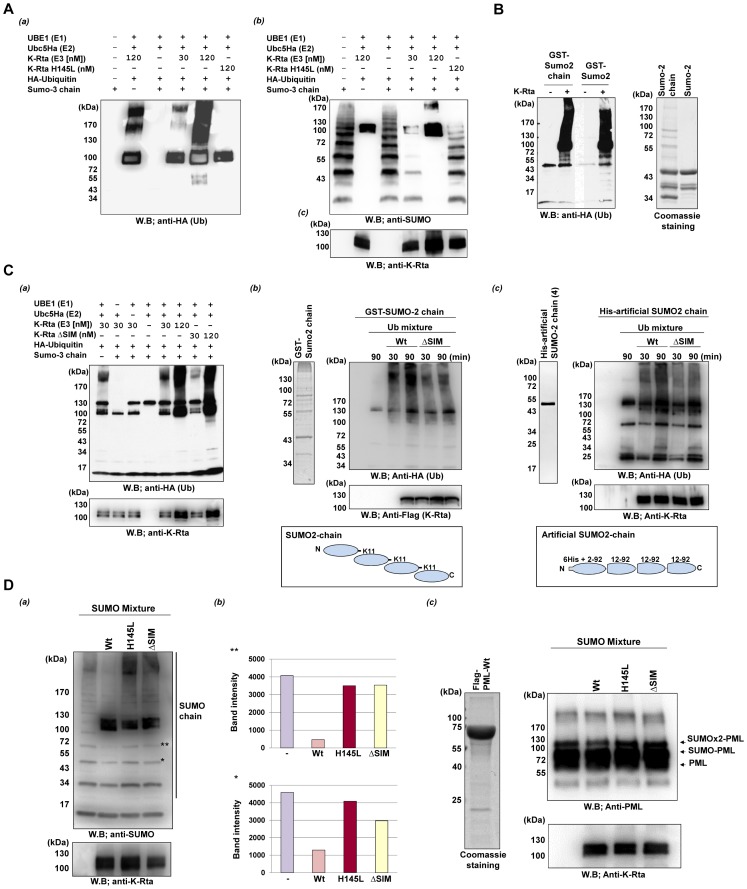
K-Rta is a SUMO-targeting ubiquitin ligase. In vitro ubiquitylation reactions were reconstituted with purified E1 (Ube1), E2 (UbcH5a), E3 (K-Rta) and HA-Ubiquitin. (**A**) SUMO-chains ubiquitylation. (a) Ubiquitylation was examined with anti-HA antibody. SUMO-chain and K-Rta were probed with anti-SUMO and anti-K-Rta antibodies, respectively (b, c). (**B**) In vitro ubiquitin reactions were performed using GST- SUMO-2 or GST-SUMO-2 chain as substrates. Purified GST-SUMO-2 and GST-SUMO-2 chain used in the reaction is shown in right panel. After ubiquitylation reactions, GST- SUMO beads were washed with high salt buffer (500 mM NaCl) containing 10% glycerol to eliminate any interacting protein. Covalently attached protein remained bound to GST-SUMO beads. The ubiquitylation reaction products were probed with an anti-HA antibody. Both SUMO and SUMO-chains were ubiquitylated only in the presence of K-Rta. **(C)** In vitro ubiquitylation reaction with K-Rta ΔSIM. (a) Ubiquitylation was examined with anti-HA antibody. The amount of K-Rta used in the reaction was shown with anti-K-Rta antibody. (b, c) The GST-SUMO-2 chain or His tagged artificial SUMO-2 chain was prepared and used as a substrate (left panels). Schematic diagram of SUMO-substrates is shown in bottom panels. Time course experiment was performed to examine efficacy of ubiquitinylation. The SUMO-substrates were isolated from the reaction at indicated time points by affinity resins, and ubiquitylation was examined by immunoblotting with anti-HA antibody. Small aliquots of reaction were taken from the reactions before isolation of SUMO-substrates and were used to confirm the amount of K-Rta in the reaction. (D) Inhibition of SUMO-conjugation in presence of K-Rta-Wt. *In vitro* SUMO-conjugation reactions were reconstituted and SUMO-2 was used as a substrate. SUMO mixture contains purified E1 (SAE1/2; 25 nM), E2 (Ubc9; 50 nM), 1 m ATP, and 2 µg of His-SUMO-2. Indicated antibodies were used to probe SUMO and K-Rta in the reaction (a). The intensity of the indicated bands was measured with image J program (b). (c) Purified PML protein was used as a substrate for SUMO-conjugation reaction. Ten microgram of purified PML protein was subjected to SDS-PAGE and stained with Coomassie (left panel). Indicated antibodies were used to probe PML and K-Rta in the reaction.

All of the data above strongly suggest that K-Rta possesses STUbL-like function. In order to demonstrate that K-Rta has an E3-ligase function toward SUMO, an *in vitro* ubiquitylation reaction was reconstituted using purified ubiquitin-related enzymes and K-Rta. Purified K-Rta Wt or mutant protein were prepared from recombinant baculovirus infected cells, and used as an E3 for ubiquitin conjugation reactions. After terminating the reaction by adding sample buffer, samples were divided onto three gels, and subjected to immunoblotting. As shown in Figure 4A(a), in the presence of the full complement of ubiquitin reaction components with K-Rta (120 nM) but not with Ring-mutant, SUMO-3 chains were effectively ubiquitylated. The observed ubiquitylation may not be due to auto-ubiquitylation of K-Rta, because significantly less ubiquitin chain formation was detected in absence of SUMO-chain (compare lane 2 vs. lane 5). This interpretation is also suggested by increased molecular weights of SUMO chains in presence of K-Rta (Figure 4A (b)). Importantly, we noticed that induction of ubiquitylation required at least 120 nM of K-Rta, which may be due to low affinity between SUMO and SIM in general [66], [71], [72]. To further ensure that the detected species is SUMO itself but not other components (e.g., K-Rta, Ub E1 and E2), the experiment was carried out with GST-SUMO-2 or GST-SUMO-2 chain conjugated beads as a substrate (Figure 4B right panel). After completion of the ubiquitin reaction, the beads were washed with a high salt buffer (500 mM NaCl) containing 10% glycerol to remove interacting proteins, and GST-SUMO-2 was eluted by boiling in sample buffer. With this approach, ubiquitin covalently attached to GST-SUMO could be effectively analyzed. Because GST itself forms a dimer, GST-SUMO-2 may be effectively a di-SUMO substrate. Nevertheless, our result clearly showed that GST-SUMO-2 and GST-SUMO-2 chains are targets of K-Rta-mediated ubiquitylation (Figure 4B left panel). As a next task, we examined the significance of K-Rta SIM domain in SUMO ubiquitylation. K-Rta Wt or K-Rta ΔSIM was used as E3's, and ubiquitylation of SUMO chains was similarly examined. Although K-Rta ΔSIM showed slightly decreased ubiquitin conjugation activity when we used lower amounts of K-Rta (Figure 4C lane 5 vs. 7), the mutant still efficiently conjugates ubiquitin to SUMO-chain with the concentration of 120 nM (Figure 4C lane 6 vs. 8). These results indicated that STUbL like function of K-Rta was not totally SIM domain-dependent *in vitro*. To further confirm the observation, two SUMO-chain substrates were prepared (Figure 4C (b, c) left panel), and the efficacy of ubiquitylation between K-Rta Wt and K-Rta ΔSIM was compared. The SUMO chain substrates were incubated in ubiquitin conjugation reaction mixture for 30 or 90 minutes. A small fraction of reaction mixture was taken to confirm equal amounts of K-Rta in each reaction. The substrates were captured by glutathione or nickel beads and the beads were washed with high salt buffer. The ubiquitylated SUMO-chain was then examined by immunoblotting (Figure 4C (b,c), right panel). The results showed that K-Rta Wt more effectively conjugated ubiquitin to SUMO-chain than the K-Rta ΔSIM, although K-Rta ΔSIM still could conjugate ubiquitin to SUMO substrates. These results of *in vitro* reactions significantly differed from co-transfection experiments (Figure 1–3) that showed nearly a complete loss of generation of SUMO conjugates with K-Rta Wt but not K-Rta ΔSIM. Thus, we decided to examine if K-Rta also inhibits conjugation of SUMO similar to chicken adenovirus Gam1 [25]. The SUMO conjugation reactions were performed in the presence or absence of K-Rta proteins, and SUMO-chains were probed with anti-SUMO antibody. The results showed that K-Rta Wt but not mutant proteins inhibited the generation of SUMO-chains *in vitro* (Figure 4D (a, b)). The fact that the reactions containing K-Rta mutants showed more SUMO conjugates may indicate that K-Rta could be a substrate of sumoylation itself, when its Ring or SIM domains are mutated. To further expand this observation, we also included PML as a substrate and performed similar experiments. After the SUMO-reaction, SUMO-modification of PML was examined by immunblotting with anti-PML antibody. The results showed again, SUMO-conjugation was inhibited in presence of K-Rta Wt (Figure 4D (c)). Taken together, these results indicated that KSHV K-Rta utilized at least two strategies to inhibit accumulation of SUMO conjugates, one for degradation of pre-existing SUMO-chains, and the other is inhibition of new SUMO chain synthesis.

### K-Rta ubiquitin ligase preferentially degrades sumoylated protein

**Figure 5:**
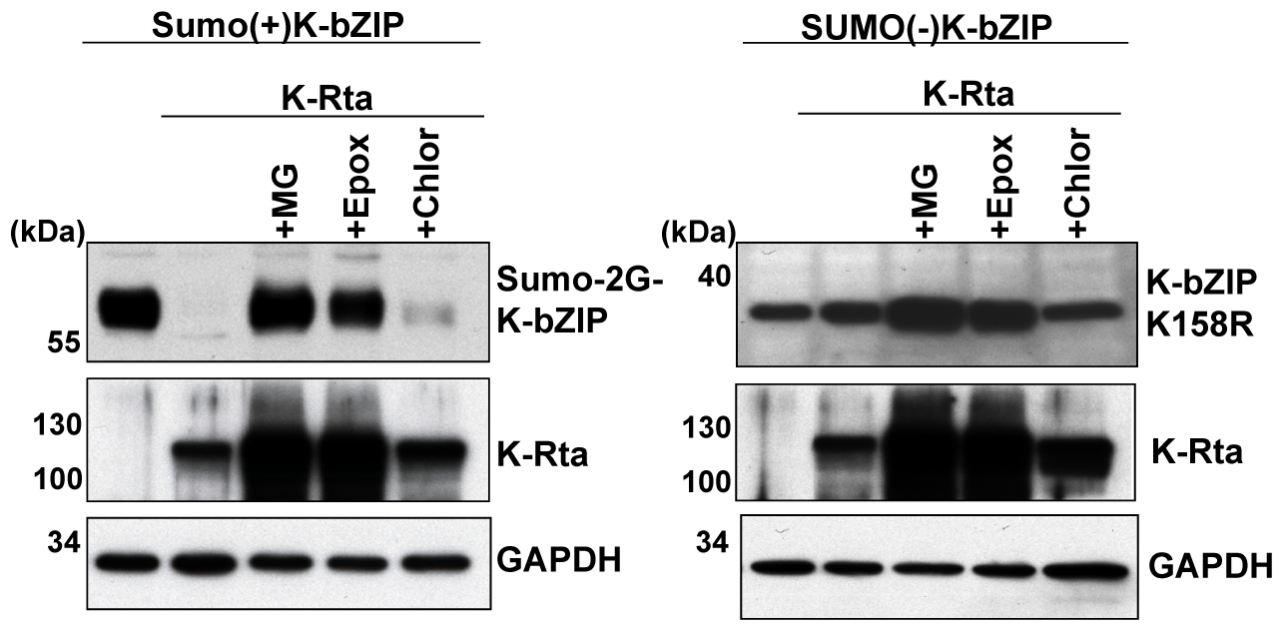
K-Rta degrades SUMO(+)K-bZIP but not SUMO(−)K-bZIP. K-Rta was cotransfected with the indicated K-bZIP plasmid and immunoblotting was performed with anti-K-bZIP antibody. Proteasome (MG132, MG; Epoxomicin, Epox) or lysosome inhibitors (chloroquine, Chlor) were added to the culture media after 24 hours transfection and incubated another 12 hours.

As a STUbL-like protein, K-Rta should catalyze the degradation of SUMO-modified proteins more effectively than non-modified species. As a next task, we examined if K-Rta preferentially targets sumoylated proteins for degradation. For this purpose, we chose K-bZIP, a KSHV encoded transcriptional repressor and an interacting protein of K-Rta. K-bZIP is sumoylated (39), and serves as a substrate for K-Rta-mediated degradation [69]. We previously showed that K-bZIP lysine 158 is the major sumoylation site [20]. To facilitate the mechanistic studies, two key K-bZIP mutants, SUMO(+) and SUMO(−), were generated. The SUMO(+) mutant was made by constructing an in-frame fusion of SUMO-2 to the N-terminus of K-bZIP, so that 100% of K-bZIP is sumoylated. The SUMO(−) mutant was made by converting residue K158, the major SUMO- acceptor site of K-bZIP, to arginine. As K-bZIP forms oligomers, this artificial substrate may present as a SUMO-chain. We examined if additional binding sites provided by SUMO facilitates degradation by the K-Rta. These mutants were transfected individually with K-Rta, and the level of engineered K-bZIP was monitored by immunoblotting using the epitope tag antibody. As shown in Figure 5, left panel, the SUMO(+) K-bZIP is rapidly degraded in the presence of K-Rta to nearly undetectable levels, and this degradation is abolished by treatment with proteasome inhibitors MG132 or epoxomicin, but not by treatment with a lysosomal inhibitor, chloroquine. In contrast, the SUMO(−) K-bZIP remains stable in the presence of K-Rta (Figure 5, right panel). These results suggest that K-Rta may preferentially target sumoylated proteins for degradation.

### K-Rta degrades sumoylated PML

**Figure 6:**
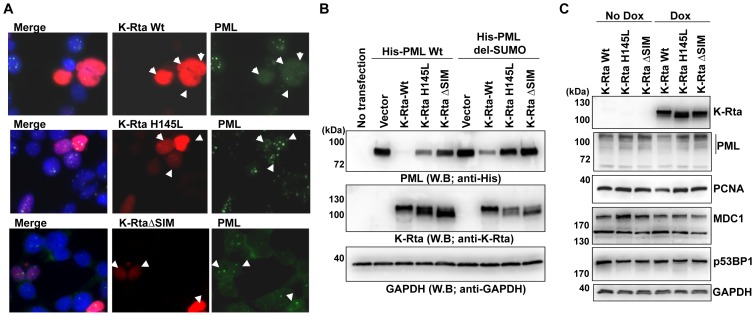
Immunofluorescence analysis (IFA). ** (A)** IFA was performed with anti-PML (Green) and anti-K-Rta (Red) antibodies. Arrows indicate cells showing overexpression of K-Rta. **(B)** K-Rta degrades PML. PML wild type or PML del-SUMO mutant was cotransfected with K-Rta or K-Rta mutants. K-Rta wild type preferentially degrades PML wild type, which can be modified by SUMO in vivo. K-Rta Ring mutant (H145L) or SUMO-binding mutant (*Δ*SIM) was not able to degrade PML. **(C)** Endogenous SUMO-modified proteins. K-Rta expression was induced by addition of doxycycline (Dox). Cell lysates were prepared 48 hours post-induction. Indicated proteins were probed with specific antibodies and 25 µg of total protein was loaded in each lane. GAPDH was used as a loading control.

As a second example of K-Rta mediated degradation of sumoylated proteins, we chose PML as a substrate. PML-NBs are “storage” sites for SUMO, and their assembly depends on the sumoylation of PML and other components such as Daxx, SP100 and HDAC [38]–[41]. In a SUMO-rich environment, sumoylated PML continues to “pull” non-sumoylated PML into the PML-NB, via SIM and dimer formation. If K-Rta has STUbL-like function, its expression should disrupt the PML-NBs, and this disruption should be dependent on the ubiquitin ligase function of K-Rta. To examine this possibility, immunofluorescence analysis was performed after transfection of K-Rta into 293T cells. The K-Rta Wt or K-Rta mutants were transfected into 293T cells, and PML and K-Rta were stained with specific antibodies. Overexpression of K-Rta Wt but not the K-Rta mutants disrupted PML-NB formation (Figure 6A). To further demonstrate that this was caused by degradation of sumoylated PML, the following experiments were carried out, based on co-transfection of PML, K-Rta and their mutant constructs. We first demonstrated that the level of ectopically expressed wt-PML was barely detectable, in the company of K-Rta Wt (Figure 6B). The PML level was much less affected by the presence of K-Rta Ring-domain mutant (K-Rta H145L) or K-Rta ΔSIM, indicating that the degradation requires both SUMO-targeting and ubiqutin ligase functions of K-Rta. By contrast, the PML-ΔSUMO mutant where the three major SUMO acceptor sites were mutated (K60R, K160R, K480R) showed much more resistance to such degradation. The observation that the PMLΔSUMO mutant still showed signs of degradation suggested that either there are other sumoylation sites of PML or the SUMO-targeting function of K-Rta is not absolutely required for degradation of SUMO-modified proteins, as is the case for other STUbL proteins [73]. Surprisingly, RNA levels of both endogenous and exogenous PML showed decreased PML expression by K-Rta Wt (Figure S4); this may also account for drastic reduction of PML in presence of K-Rta Wt. Finally, effects on endogenous proteins that are known to be modified by SUMO were examined with K-Rta inducible cells. K-Rta Wt or mutant inducible cells were generated with TREx-293 cells by cotransfection with Flp recombinase. After induction of K-Rta with doxycycline, endogenous proteins were probed with specific antibodies. The results showed that K-Rta-Wt, but not mutant, reduced PML and PCNA expression but did not affect expression of MDC1 or p53BP1. The results that endogenous PML is degraded by K-Rta much less efficiently than exogenous PML may suggest that a higher concentration of SUMO-modified protein may be necessary in order for K-Rta to recognize as substrates. Taken together, K-Rta preferentially targets PML Wt over PML ΔSUMO for degradation, and the degree of SUMO modification and concentration of substrates may determine the outcome of K-Rta mediated degradation.

### K-Rta ubiquitin ligase function is important for KSHV promoter activation, gene expression, and viral replication

**Figure 7:**
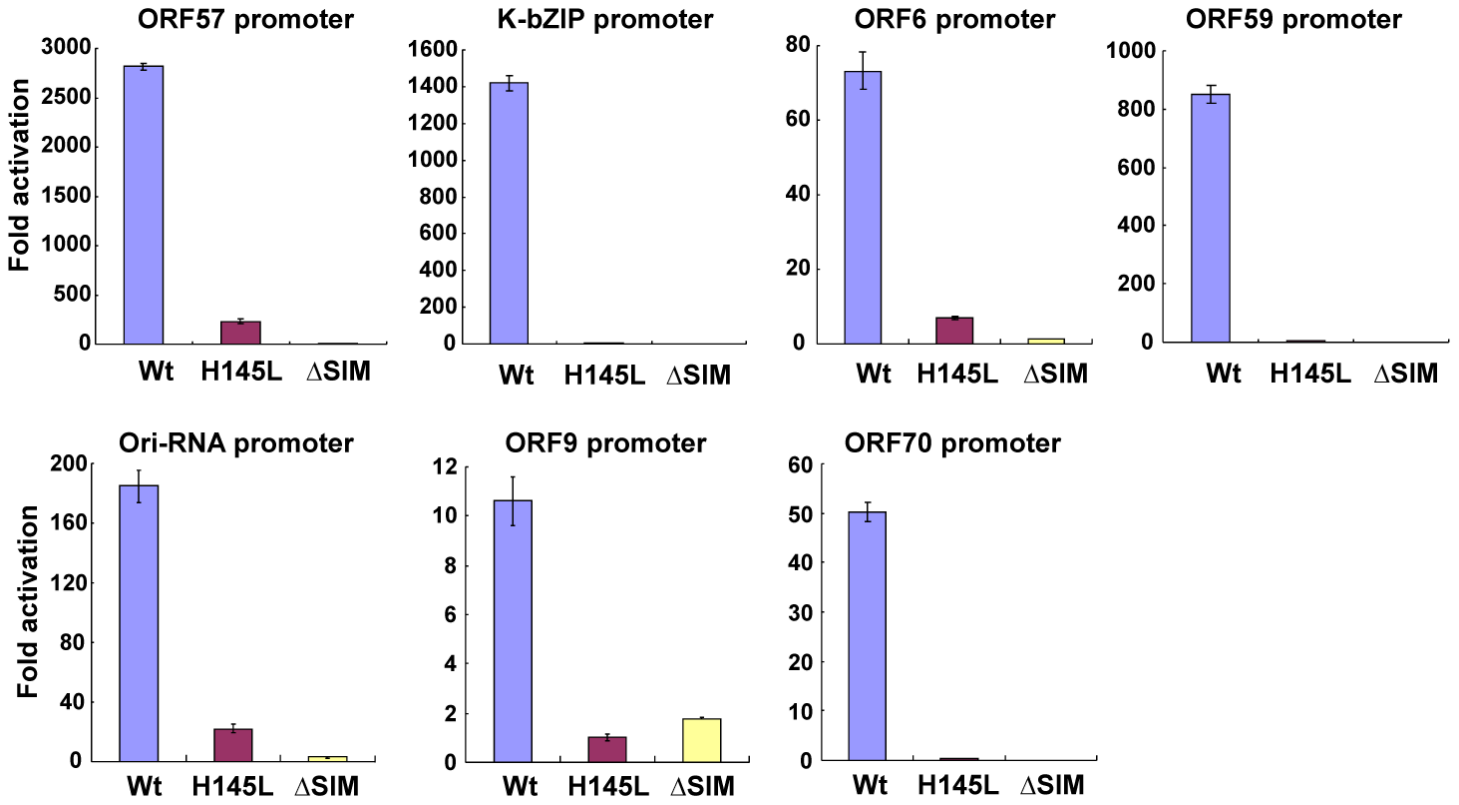
STUbL-like function is important for K-Rta transactivation activity. Reporter assays were performed in 293 cells using the indicated K-Rta target gene reporters. Reporter plasmids were cotransfected with K-Rta Wt or mutants, and luciferase activity was measured at 48 hours post-transfection. Luciferase activity of reporter with empty expression plasmids was normalized to a value of 1. Fold activation over control is shown.

**Figure 8:**
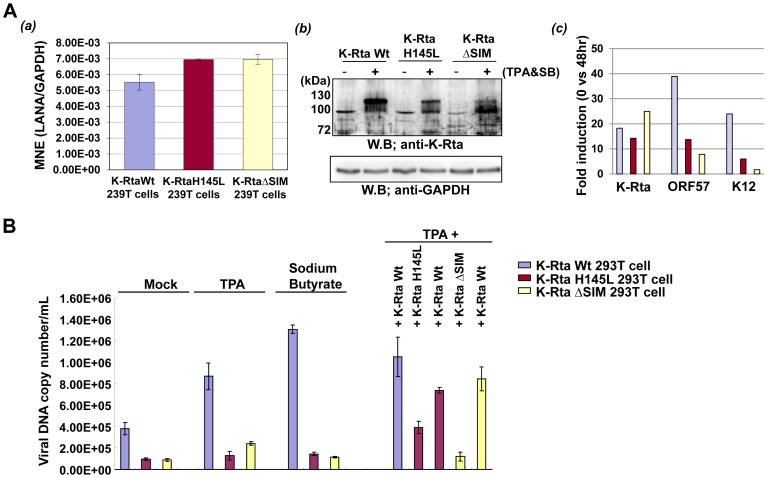
SUMO-degradation function is important for KSHV replication. **(A)** Recombinant KSHV, which harbors a point mutation in the Ring domain of K-Rta or SIM domain was constructed and transfected into 293T cells. LANA expression was examined by RT-qPCR (a). Values are normalized to cellular GAPDH. Recombinant KSHV containing a K-Rta point mutation or revertant wild type virus was reactivated with combination of sodium butyrate (SB, 1 mM) and TPA (20 nM). Expression of K-Rta protein was confirmed by immunoblotting (b), and K-Rta target gene expression was examined by RT-qPCR (c). Values are normalized to cellular β-actin, and fold induction over latent cell is shown. **(B)** Viral replication. Recombinant KSHV replication was measured after reactivation with TPA, sodium butyrate, or combination of K-Rta expression and TPA treatment. KSHV encapsidated (DNase resistant) DNA copy number in the supernatant was measured by qt-PCR. Absolute viral copy number in 1 mL of supernatant is shown. K-Rta Wt but not mutant expression rescued viral replication.

A large body of evidence suggests that SUMO-modification is associated with gene repression. For the next task, we asked whether K-Rta's ability to remove SUMO and sumoylated proteins is relevant to its transactivation functions and the ability to activate viral replication. To this end, we took advantage of the ubiquitin ligase deficient K-Rta H145L mutant and SIM mutant, which are unable to degrade SUMO. As shown in Figure 7, the ORF57 promoter, a well-recognized promoter transactivated by K-Rta, is activated by the wild type K-Rta, but to a much less extent by the H145L or K-Rta ΔSIM mutant. Similar results were also obtained with 6 other KSHV promoters. To expand on this observation in regards to viral reactivation, a recombinant KSHV knock-in mutant encoding a K-Rta Ring-finger mutation or SIM mutation was generated with KSHV BAC16. The scheme of generation and verification of the knock-in mutant is described in Figure S1A. A revertant virus was generated from the Ring-finger mutant by repeating the same recombination procedures with a K-Rta wild type targeting DNA fragment. Recombinant BACs were transfected into 293T cells to establish stable cells. After generating stable 293T cells harboring recombinant KSHV (Figure S1B), we examined the expression pattern of K-Rta target genes after TPA stimulation. To examine possible copy number variation among recombinant BAC stable cells, we also examined LANA expression, which is an indicator of KSHV episome copy number. The results showed that K-Rta Wt cells showed slightly lower LANA expression, but two mutant stable cells showed very similar expression (Figure 8A(a)). Using these cells, we next examined lytic gene expression after reactivation with a combination of TPA and sodium butyrate. The cells were incubated with TPA and sodium butyrate for 24 hours and K-Rta protein expression as well as lytic gene expression was examined. Although K-Rta ΔSIM expression was higher when compared with K-Rta Wt stable cells, K-Rta ΔSIM mutant could not activate downstream target genes such as ORF57 and K12 (Figure 8A(c) and Figure S5). Similarly, the K-Rta Ring-mutation also significantly impaired K-Rta transactivation function. As a next step, viral replication was examined by measuring viral copy number in the supernatant after reactivation. TPA or sodium butyrate was added to the culture media and viral copy number in the supernatant was measured at 5 days post-stimulation. As shown in Figure 8B, K-Rta Wt showed much higher viral copy numbers even in absence of stimulation, which may be due to spontaneous reactivation. The mutation in the Ring-finger like domain or SIM domain significantly abolished viral reactivation/replication. To confirm that the observed phenotype was due to the introduced mutation, a K-Rta Wt expression plasmid was transfected into that mutant cells in order to rescue the phenotype. TPA was also added into culture media to stimulate viral reactivation. The results showed that K-Rta Wt but not mutant rescued viral replication (Figure 8B). These results clearly suggested that both the Ring-finger domain and SIM domain are crucial for K-Rta function(s) to reactivate virus.

### Sumoylation inhibits KSHV gene expression

**Figure 9:**
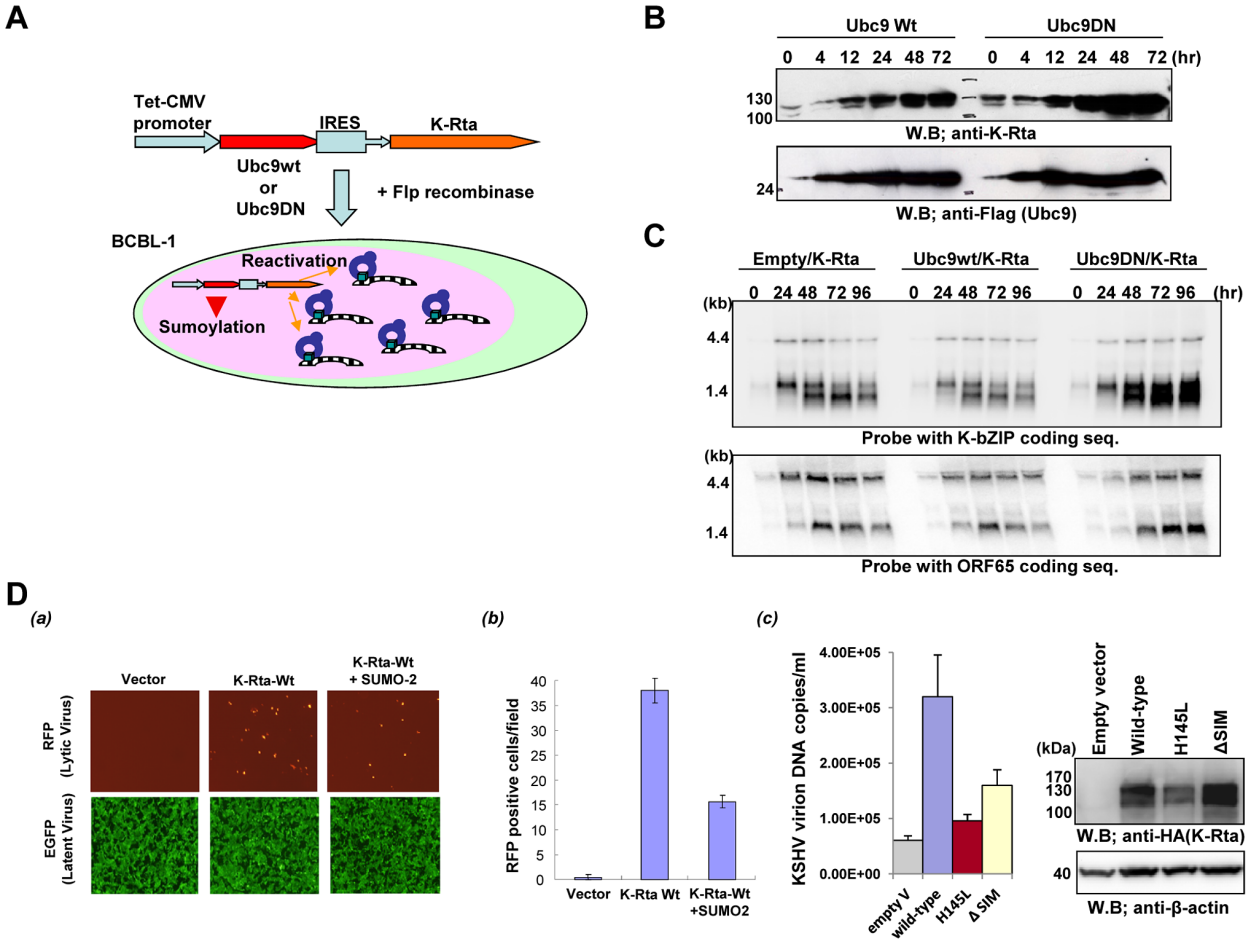
Sumo inhibits KSHV gene expression and reactivation. **(A)** A dual-inducible cell line was generated with the TREx FRT recombination system. Indicated protein was probed with specific antibodies **(B)**, and gene expression was examined by northern blotting with probes to the coding regions of K-bZIP or ORF65 **(C)**. Ubc9 wild type decreased gene expression; however, the Ubc9 mutant increased viral gene expression compared to control cells. **(D)** SUMO inhibits viral reactivation. Indicated plasmids were cotransfected into recombinant KSHV infected Vero cells. (a) RFP positive cells were counted 72 hours after transfection in five randomly selected fields. (b) Average of RFP positive cells are shown. The total amount of plasmid/well was kept at 2 micrograms by adding control vector DNA. (c) Viral copy number after transfection of K-Rta or mutants into recombinant KSHV infected Vero cells is shown. Supernatants were collected 5 days post-transfection and encapsidated viral DNA was measured by qt-PCR (left panel). K-Rta expression was confirmed by immunoblotting (right panel).

Finally, we examined the overall effects of the SUMO-pathway in viral lytic replication. Inducible BCBL-1 cells were engineered to express both SUMO-E2 (Ubc9wt or mutant Ubc9C93S) and K-Rta from the same mRNA by inserting an IRES site between these two genes in the expression cassette. The expression cassettes were inserted in the same genomic locus by FLPE mediated recombination and driven by the same CMV promoter, which allows us to compare between two cell lines (Figure 9A). After inducing reactivation by K-Rta with doxycycline, total cell protein and RNA were harvested, and viral gene expression was examined. Induction of K-Rta and Ubc9 were confirmed by immunoblotting (Figure 9B). Downstream viral gene expression was then examined by northern blotting (Figure 9C). As expected, overexpression of Ubc9-Wt inhibits KSHV gene expression, exhibiting viral gene expression profiles which decline earlier than in the control cells. On the other hand, Ubc9C93S significantly enhanced and sustained viral gene expression compared to the vector control cells. These results indicated that SUMO modification may be important for shutting down KSHV gene expression, which is consistent with observations made in HSV-1 infected cells [55], [74]. To further examine and complement these observations, another approach was taken to examine effects of SUMO on viral gene expression. The SUMO and K-Rta-Wt were co-transfected into recombinant KSHV-infected Vero cells, and reactivation was monitored by RFP (red fluorescence protein) positive cells; expression of RFP is controlled by a viral lytic promoter (PAN). Five different microscopic fields were counted for RFP positive cells. As shown in Figure 9D, if a SUMO-2 expression plasmid was co-transfected with K-Rta wild type, KSHV reactivation was decreased 2.5-fold relative to the reactivation level achieved by transfection of K-Rta alone (Figure 9D (a, b)). Similarly, mutation in the Ring-domain or SIM domain significantly decreased K-Rta's ability to reactivate KSHV (Figure 9D (c)). The amount of exogenous K-Rta expression was confirmed by immunoblotting with anti-HA antibody (Figure 9D (c)). Taken together, these results indicated that the SUMO-pathway plays an inhibitory role in KSHV gene expression, and K-Rta STUbL-like function may counteract SUMO-mediated gene repression to sustain viral gene expression.

## Discussion

Previously, we showed that the K-bZIP protein of KSHV is a SUMO-ligase [75]. In this report, we show that KSHV encodes a SUMO targeting ubiquitin ligase. To our knowledge, this is the first example of a virus which has evolved such a strategy to dynamically regulate the host SUMO environment. Changing the SUMO environment represents an effective and reversible way of regulating viral and host transcriptional programs. In general, SUMO-rich environments favor gene silencing, whereas a SUMO-free environment is more transcriptionally active. For instance, the formation of KAP-1/HP-1 complex which characterizes heterochromatinization and decorates chromosomes with repressive H3K9me3 marks is SUMO-dependent [66]. Likewise, the recruitment of global gene silencer, the polycomb 2 complex (e.g., Pc2/CBX4) to H3K27me3 repressive marks is also enhanced by SUMO modification [76]. The ability of K-Rta to degrade SUMO and SUMOylated proteins adds to its prowess as a transactivator, and contributes to its potency in triggering the reactivation of latent viral genomes. This is consistent with the reports by us and by others that the latent KSHV genome is “coated” with KAP-1 and decorated with H3K27me3 and H3K9me3 repressive marks [61], [64], [65]. Upon K-Rta expression, the viral chromatin showed lowered levels of these repressive marks and is associated with hyper-acetylated histones [61], [64], [65]. Further work is required to confirm the detailed role of SUMO in KSHV reactivation.

Disassembly of PML bodies early after infection is a common characteristic of herpesviruses. Different viruses utilize different mechanisms to achieve this goal [22], [25], [58], [60]. We provide data showing K-Rta's ability to disperse PML bodies and degrade PML. The degradation of PML is largely SUMO-dependent, as K-RtaΔSIM mutants are much less effective in the degradation of PML. Moreover, a PML mutant with the major SUMOylation sites mutated is more resistant to such degradation. Although K-Rta has a preference on SUMOylated PML, we found that both SUMOylated and un-sumoylated PMLs are depleted in the presence of K-Rta. There are three possible explanations for this result: 1) The requirement of SUMO for K-Rta's ubiquitin ligase activity is not absolute, 2) PML is constantly being sumoylated by cellular E3 ligases, 3) SUMOylated PML continuously recruits (or pulls) non-SUMO modified PML via coiled-coil domain resulting in degradation of both species. The fact that K-Rta Ring-mutant still degrades PML in transfected cells indicates that there may be alternative pathways for PML degradation employed by K-Rta. It has been reported that K-Rta interacts with the HECT E3 ligase, RAUL and counteracts the IFN response [77]. Future studies are required to determine if RAUL can target PML protein for degradation.

Our studies showed that the SIM domain of K-Rta is not essential for ubiquitylation of SUMO *in vitro*. It has been shown that STUbLs often act relatively non-specifically, ubiquitylating artificial substrates such as GST-SUMO and free poly-SUMO chains [68], [73], [78]. In addition, SUMO-independent protein degradation by STUbLs has also been reported [79], [80]. These facts suggest that SUMOylation may provide additional binding sites that facilitate protein degradation by STUbLs.

Using the cellular DNA damage response as an example, Psakhye et al [81] recently proposed a new concept of SUMO modification, in which sumoylation targets protein groups rather than individual proteins, with SUMO protein acting as “molecular glue” to assemble protein complexes as needed to carry out DNA repair events. It is tempting to speculate that herpesviruses may possess a “remover” to counteract host cell protein assembly required for antiviral responses.

In addition to STUbL-like function, we noticed that K-Rta could inhibit the generation of SUMO-chains *in vitro*. Importantly, the inhibition of SUMO-chain formation is both SIM and Ring-finger like domain dependent. A recent study showed that Ring-finger domain of RNF4 interacts with Ub-loaded E2 with higher affinity [82]. It is possible that K-Rta may recognize SUMO-loaded Ubc9 and bind through SIM and Ring-finger like domains, which may further inhibit formation of SUMO-chain. Further studies are required for revealing detailed mechanisms.

A recent study showed that re-stimulation of cells with IFN-gamma leads to earlier and stronger MHCII transcription induction, which is attributed to persistent levels of H3K4me2. Interestingly, this epigenetic change correlates with re-localization of the genomic locus that moves to the proximity of PML-NBs, which have been shown to have a direct role in the persistence of memory and H3K4me2 levels in the promoter, because depletion of PML compromises the maintenance of H3K4me2 and magnitude of memory responses [83]. This mechanism of transcriptional memory may be applied to herpesviruses gene expression, because a large body of evidence suggests that herpesvirus gene expression and DNA replication occurs in the proximity of PML-NBs at later stages of infection [84]–[90]. If this is the case, K-Rta may initiate reorganization of PML-NBs by disrupting pre-existing PML-NBs. It will be important to determine if PML-NBs are formed on the KSHV genome as observed with HSV-1 [55].

An additional interesting finding from this study is the antagonism between K-Rta and K-bZIP. Previously, we showed that the activity of K-Rta is inhibited by K-bZIP through direct association. Now we show that K-Rta in turn can degrade SUMO-K-bZIP, the repressive form of K-bZIP. Another functional antagonism between these two early gene products is noted as K-Rta decreases the cellular SUMO environment, whereas K-bZIP increases it. This is perhaps necessary for maintaining temporal regulation of the early and late genes during lytic replication, as well as the decision to enter latency. RNF4, which contains multiple SIMs and recognizes poly-SUMO proteins, is presently the only STUbL identified for human cells. Since only SUMO-2 and -3 are able to form chain structures, the substrates of RNF4 are mostly modified by SUMO-2/3. The study by Ron Hay and his colleagues showed that RNF4 preferentially degrades SUMO-2/3 modified PML as compared to SUMO1-PML [57]. In the case of K-Rta, this viral STUbL-like protein seems to prefer SUMO-2/3 multimers as well, but also recognizes SUMO-1 targets, at least when SUMO is overexpressed in cells. Thus far, we and others report that K-Rta targets IRF7, K-RAB, K-bZIP, and LANA for degradation [69], [70]. LANA is heavily sumoylated and the degradation of LANA by K-Rta clearly will facilitate the reactivation from latent genomes. Accordingly, K-Rta Ring-finger or SIM mutant has much lower transactivation activity, which is accompanied by a reduction in viral replication. In addition to LANA, there are other potential targets of K-Rta, such as Daxx and KAP1, whose degradation will likely prepare suitable environment for reactivation and replication. These proteins are known to be heavily sumoylated and their sumoylated forms serve to transcriptionally repress or assemble condensed chromatin around the viral genome [61], [91]. Consistent with this, overexpression of SUMO or Ubc9 represses KSHV gene expression in the course of KSHV reactivation (Figure 9).

In the course of generating K-RtaΔSIM mutants, we noticed that all K-Rta mutants which were impaired in their STUbL-like function were less phosphorylated in transfected cells. Interestingly, a recent study on HSV-1 ICP0 demonstrated that phosphorylation of ICP0 by CK1 is required for binding to RNF8 and subsequent ubiquitylation [92]. Although we do not know if phosphorylation is required for K-Rta to bind SUMO, the regulation of SUMO binding by phosphorylation has been documented elsewhere [10], [93]. It will be of particular interest to identify the kinase responsible for K-Rta phosphorylation and determine its effects on SUMO-degradation. Conversely, SUMO binding may enhance the recruitment of a kinase for the phosphorylation of K-Rta.

During herpesvirus simplex virus 1 (HSV-1) infection, components of PML-NBs are recruited to sites associated with the viral genome soon after they enter the nucleus. SUMO-modification sites and SIMs of PML, as well as Sp100 and Daxx are required for both formation of PML-NBs and recruitment to the viral genome [55]. In HSV-1 infected cells, ICP0, a viral ubiquitin ligase, preferentially degrades SUMO-modified forms of PML, which counteracts intrinsic immunity and completes lytic infection [58]. Unlike HSV-1 infection, KSHV has a tendency to enter a latency program by shutting off most of the viral genes soon after infection. This process could be facilitated by the increase of sumoylated proteins and reassembly of PML-NBs by K-bZIP or another cellular SUMO E3 ligase. Interestingly, our recent studies showed that KSHV LANA has a function to increase histone SUMO modification *in vitro* and *in vivo*, and LANA recruitment sites are largely overlapped with SUMO-rich sites on the KSHV genome [94]. These results suggest the possibility that LANA maintains the SUMO status of latent viral genomes, and the removal of SUMO by K-Rta represents one of the early steps in reactivation. We are currently investigating when and how PML-NBs are recruited to the KSHV genome, and its effects on epigenetic signatures at the recruited sites on the KSHV genome.

In summary, we have uncovered a virally encoded STUbL-like activity in KSHV and shown the critical role of SUMO-targeting and Ubiquitin ligase activities of K-Rta in viral replication. By degrading SUMO and sumoylated repressor proteins, K-Rta may effectively and globally regulate the host signaling pathways including the interferon response as well as facilitating the unfolding of condensed viral chromatin to enhance the transcription and replication of the viral genome.

## Supporting Information

Figure S1 Generation of Recombinant KSHV. (A) (a) Schematic diagram of BAC recombination. A K-Rta transfer vector was introduced into SW105 cells harboring BAC16. Kanamycin-resistant clones were isolated and recombination was confirmed by PCR screening and Southern blotting (b). After confirming correct recombination, the Kanamycin cassette was deleted by Flp recombination, thus only the 34 bp FRT fragment remained in the KSHV genome. Subsequently, the introduced point mutation in K-Rta was confirmed by direct sequencing of the PCR-amplified fragment from BAC DNA. A similar procedure was used to introduce the wild type K-Rta fragment to generate a revertant clone. (B) Establishment of recombinant BAC stable cells. Recombinant BAC16 was transfected into 293T cells and selected with hygromycin for 2 weeks. The GFP signal was confirmed by an inverted fluorescence microscope.
(TIF)

Figure S2 K-Rta targets SUMO isoforms. SUMO-1, -2, or -3 was cotransfected with K-Rta, and SUMO-modified proteins were probed with an anti-T7-tag antibody. K-Rta expression was confirmed by immunoblotting with an anti-K-Rta antibody, and equal loading was examined by probing same membrane with an anti-actin antibody.
(TIF)

Figure S3 (**A**) Mapping of K-Rta SIMs. Putative K-Rta SIMs (hydrophobic cluster) was mutated to alanine and the mutant expression plasmid was co-transfected with PML-Wt expression vector. Degradation of PML-Wt was measured with immunoblotting. K-RtaΔSIM was used as control and compared with other K-Rta mutants. K-Rta expression was also confirmed by immunoblotting (bottom panel). (**B**) GST-pull down analyses. GST-pull down analyses were performed with either K-Rta Wt or K-Rta ΔSIM mutant and compared side by side on the same gel. K-Rta ΔSIM showed less affinity to GST-SUMO-2 and GST-SUMO-3 compared with K-Rta Wt.
(TIF)

Figure S4 Transcriptional levels of endogenous or exogenous PML. The cDNA of endogenous PML or exogenous PML was synthesized with specific primers (PML specific primers for endogenous PML, a BGH-reverse primer for exogenous PML). The qt-PCR was used to measure transcripts of PML. cDNA of GAPDH generated by random hexamer oligonucleotides were used as internal control for both reactions.
(TIF)

Figure S5 (A) Viral gene expression. The 293T cells harboring recombinant KSHV were reactivated with combination of TPA and sodium butyrate (SB). Viral transcripts were normalized with cellular GAPDH and the mean normalized expression (MNE) is shown. Wt; K-Rta wild type, HL; K-Rta H145L mutant, ΔSIM; K-Rta ΔSIM mutant.
(TIF)
