# Supplementary figures and images for: Kaposi's Sarcoma-Associated Herpesvirus K-Rta Exhibits SUMO-Targeting Ubiquitin Ligase (STUbL) Like Activity and Is Essential for Viral Reactivation
Source: PLoS Pathog. 2013 Aug 22;9(8):e1003506. doi: 10.1371/journal.ppat.1003506 (PMC3749962; doi:10.1371/journal.ppat.1003506)

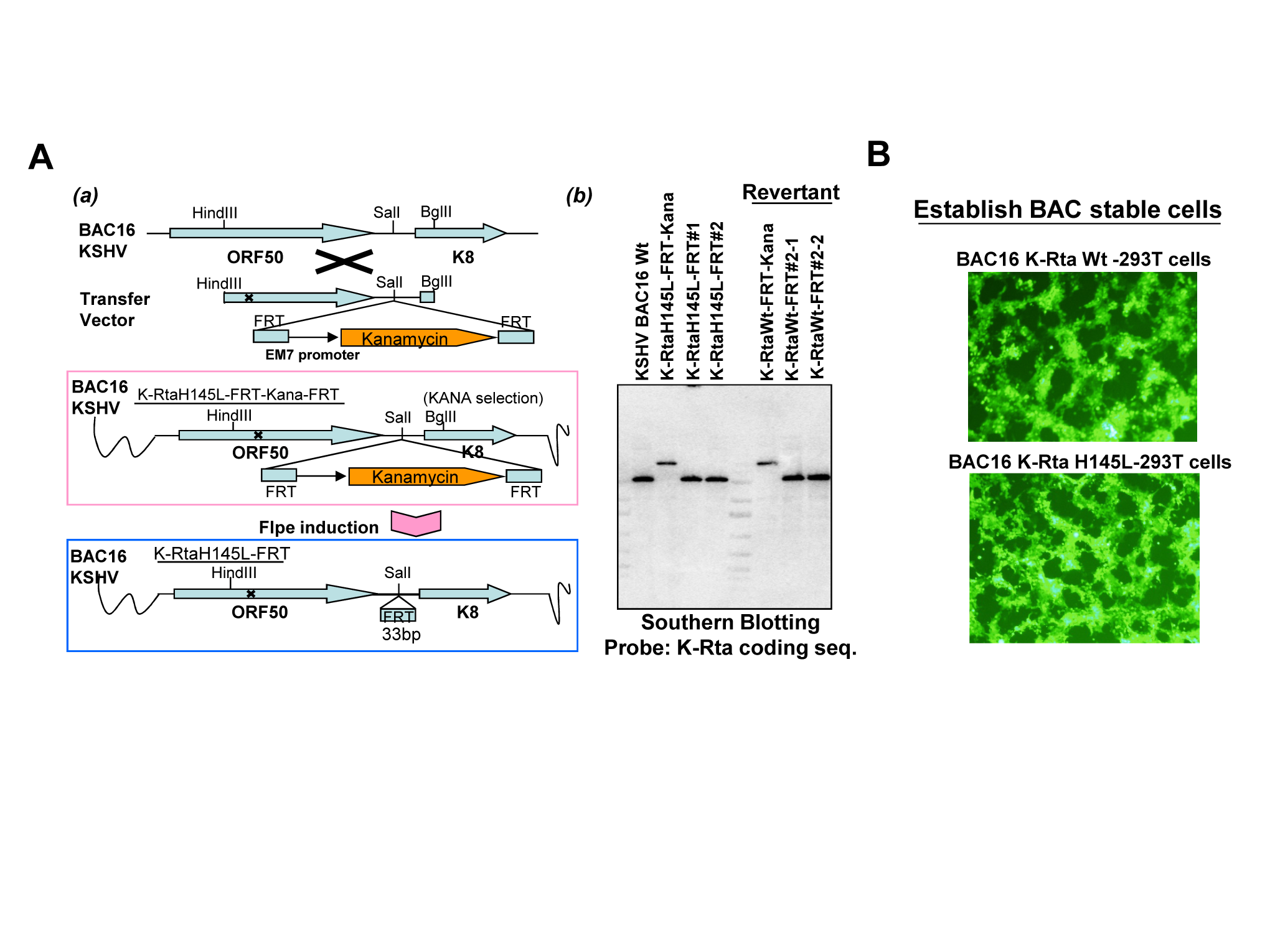

Supplement: Figure S1 — Generation of Recombinant KSHV. (A) (a) Schematic diagram of BAC recombination. A K-Rta transfer vector was introduced into SW105 cells harboring BAC16. Kanamycin-resistant clones were isolated and recombination was confirmed by PCR screening and Southern blotting (b). After confirming correct recombination, the Kanamycin cassette was deleted by Flp recombination, thus only the 34 bp FRT fragment remained in the KSHV genome. Subsequently, the introduced point mutation in K-Rta was confirmed by direct sequencing of the PCR-amplified fragment from BAC DNA. A similar procedure was used to introduce the wild type K-Rta fragment to generate a revertant clone. (B) Establishment of recombinant BAC stable cells. Recombinant BAC16 was transfected into 293T cells and selected with hygromycin for 2 weeks. The GFP signal was confirmed by an inverted fluorescence microscope. (TIF) [file ppat.1003506.s001.tif]

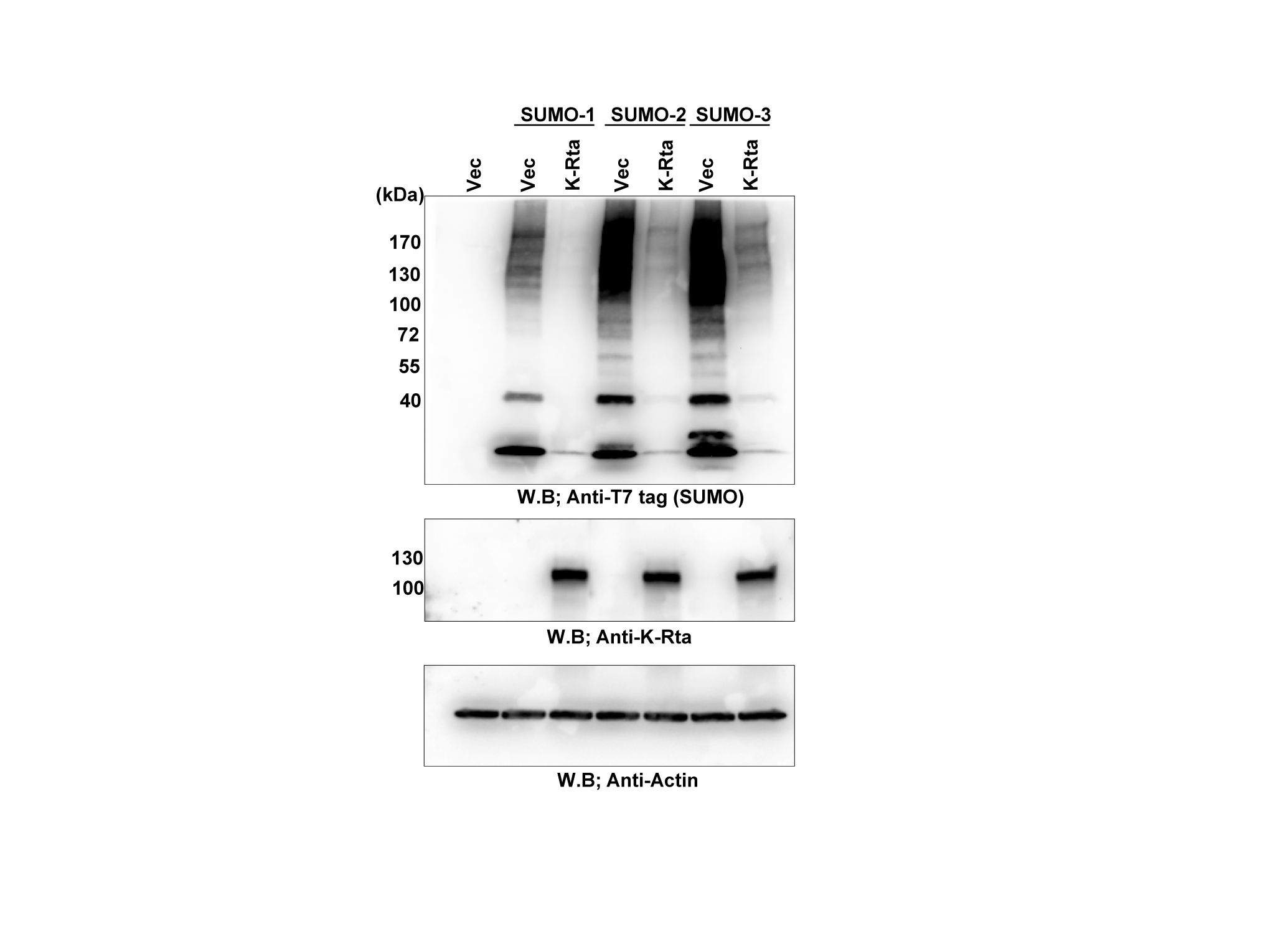

Supplement: Figure S2 — K-Rta targets SUMO isoforms. SUMO-1, -2, or -3 was cotransfected with K-Rta, and SUMO-modified proteins were probed with an anti-T7-tag antibody. K-Rta expression was confirmed by immunoblotting with an anti-K-Rta antibody, and equal loading was examined by probing same membrane with an anti-actin antibody. (TIF) [file ppat.1003506.s002.tif]

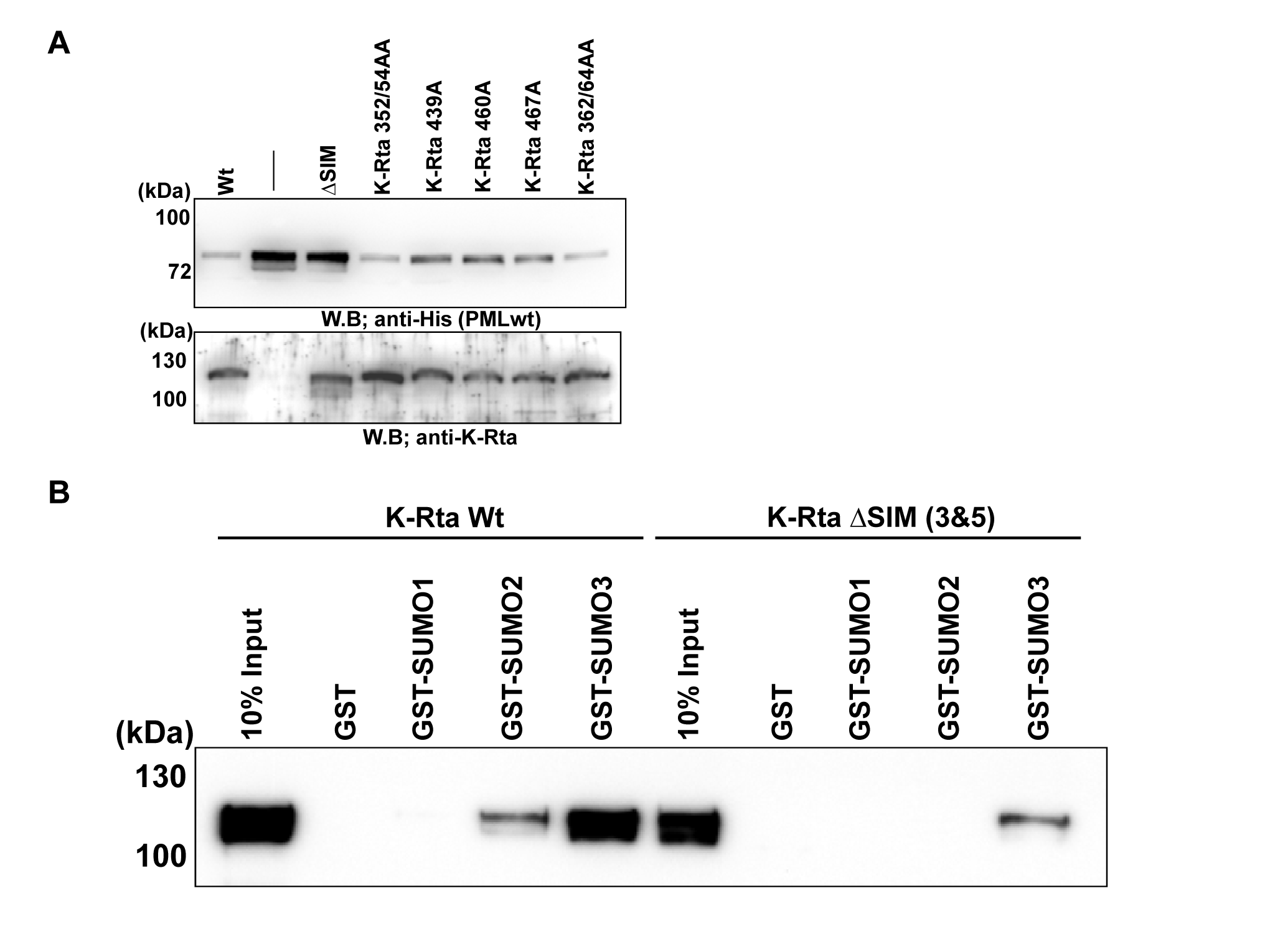

Supplement: Figure S3 — (A) Mapping of K-Rta SIMs. Putative K-Rta SIMs (hydrophobic cluster) was mutated to alanine and the mutant expression plasmid was co-transfected with PML-Wt expression vector. Degradation of PML-Wt was measured with immunoblotting. K-RtaΔSIM was used as control and compared with other K-Rta mutants. K-Rta expression was also confirmed by immunoblotting (bottom panel). (B) GST-pull down analyses. GST-pull down analyses were performed with either K-Rta Wt or K-Rta ΔSIM mutant and compared side by side on the same gel. K-Rta ΔSIM showed less affinity to GST-SUMO-2 and GST-SUMO-3 compared with K-Rta Wt. (TIF) [file ppat.1003506.s003.tif]

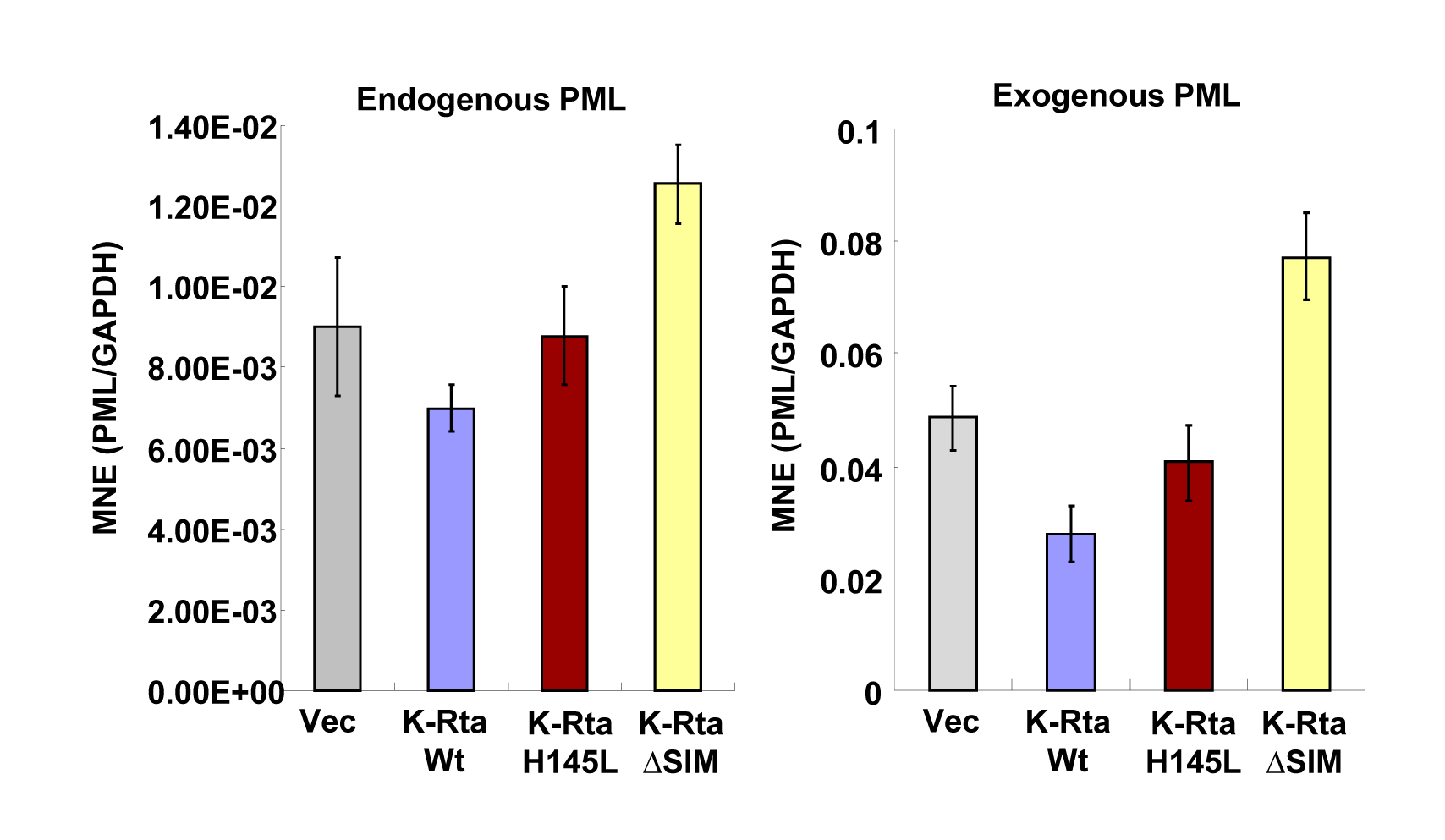

Supplement: Figure S4 — Transcriptional levels of endogenous or exogenous PML. The cDNA of endogenous PML or exogenous PML was synthesized with specific primers (PML specific primers for endogenous PML, a BGH-reverse primer for exogenous PML). The qt-PCR was used to measure transcripts of PML. cDNA of GAPDH generated by random hexamer oligonucleotides were used as internal control for both reactions. (TIF) [file ppat.1003506.s004.tif]

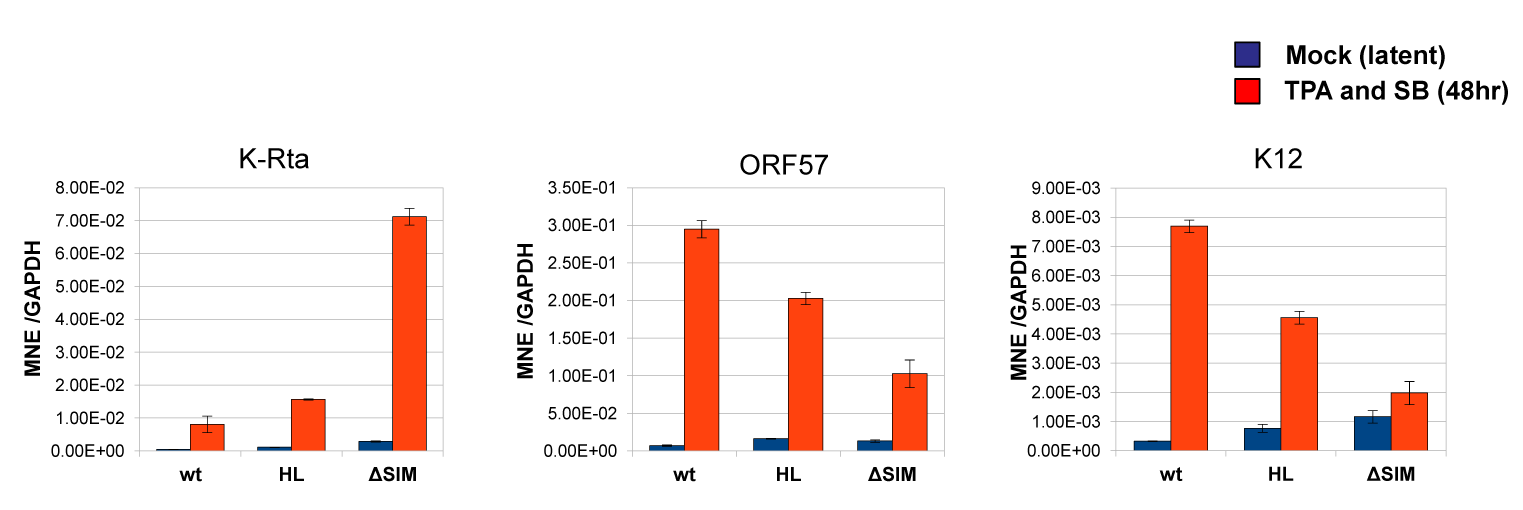

Supplement: Figure S5 — (A) Viral gene expression. The 293T cells harboring recombinant KSHV were reactivated with combination of TPA and sodium butyrate (SB). Viral transcripts were normalized with cellular GAPDH and the mean normalized expression (MNE) is shown. Wt; K-Rta wild type, HL; K-Rta H145L mutant, ΔSIM; K-Rta ΔSIM mutant. (TIF) [file ppat.1003506.s005.tif]
